# Enhancing Lung Recovery: Inhaled Poly(lactic-*co*-glycolic) Acid Encapsulating FTY720 and Nobiletin for Lipopolysaccharide-Induced Lung Injury, with Advanced Inhalation Tower Technology

**DOI:** 10.1021/acsnano.3c12532

**Published:** 2025-02-18

**Authors:** Huei-Han Zhang, Wen-Shuo Kuo, Pei-Yu Tu, Chung-Ta Lee, Hao-Chen Wang, Yu-Ting Huang, Mei-Chun Shen, Tsai-Shiuan Lin, Po-Lan Su, Jeng-Shiuan Tsai, Min-Hsiung Pan, Chien-Chung Lin, Ping-Ching Wu

**Affiliations:** a Department of Biomedical Engineering, National Cheng Kung University, Tainan 70101, Taiwan; b Center for Allergy Immunology and Microbiome (AIM), China Medical University Children’s Hospital/China Medical University Hospital, China Medical University, Taichung 404327, Taiwan; c Department of Pathology, National Cheng Kung University Hospital, College of Medicine, National Cheng Kung University, Tainan 701401, Taiwan; d Medical Imaging Center, Innovation Headquarters, National Cheng Kung University, Tainan 70101, Taiwan; e Department of Internal Medicine, National Cheng Kung University Hospital, College of Medicine, National Cheng Kung University, Tainan 70403, Taiwan; f Graduate Institute of Clinical Medicine, College of Medicine, National Cheng Kung University, Tainan 701401, Taiwan; g Institute of Food Science and Technology, National Taiwan University, Taipei 10617, Taiwan; h Department of Medical Research, China Medical University Hospital, China Medical University, Taichung 404327, Taiwan; i Tainan Hospital, Ministry of Health & Welfare, Tainan 70101, Taiwan; j Institute of Molecular Medicine, College of Medicine, National Cheng Kung University, Tainan 700, Taiwan; k Center of Applied Nanomedicine, National Cheng Kung University, Tainan 70101, Taiwan; l Medical Device Innovation Center, Taiwan Innovation Center of Medical Devices and Technology, National Cheng Kung University Hospital, National Cheng Kung University, Tainan 70403, Taiwan

**Keywords:** fingolimod (FTY720), nobiletin (NOB), inhaled nanoformulation, acute lung injury (ALI), cytokine suppression, immune cell infiltration

## Abstract

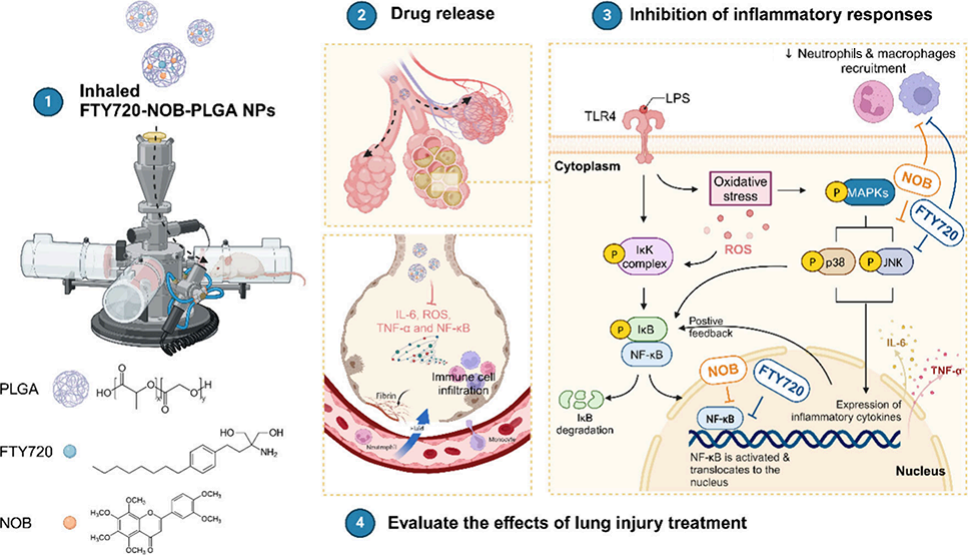

Acute lung injury (ALI)/acute respiratory distress syndrome (ARDS), a rapidly progressing respiratory failure condition, results in a high mortality rate, especially in severe cases. Numerous trials have investigated various pharmacotherapy approaches, but their effectiveness remains uncertain. Here, we present an inhaled nanoformulation of fingolimod (FTY720)-nobiletin (NOB)- poly(lactic-*co*-glycolic) acid (PLGA) nanoparticles (NPs) with good biocompatibility and a sustained-release pharmacological effect. The formulation decreases the toxicity of FTY720 and increases the bioavailability of NOB since we use PLGA with a high biocompatibility to encapsulate FTY720 and NOB at the same time. *In vitro*, in comparison to treatment with the pure drug, we demonstrated that FTY720-NOB-PLGA NPs can reduce interleukin-6 (IL-6) and reactive oxygen species (ROS) release by macrophages after lipopolysaccharide (LPS) stimulation more efficiently. *In vivo*, we used an inhalation tower system that allowed the exposure of unanesthetized mice to aerosolized FTY720-NOB-PLGA NPs under controlled conditions. We demonstrated that inhaled FTY720-NOB-PLGA NPs can attenuate lung injury after LPS exposure by suppressing cytokine release, such as IL-6 and tumor necrosis factor-α (TNF-α). The trigger pathway of ALI, including nuclear factor κ-light-chain-enhancer of activated B cells (NF-κB) and p38 mitogen-activated protein kinase, was also efficiently inhibited. Furthermore, the inhalation treatment provided a good safety profile, without detrimental effects on biochemical markers and lung function. We provided the feasibility of administering inhalation of NPs noninvasively with continuous monitoring of lung function. The aerosolized FTY720-NOB-PLGA NPs we developed show excellent promise for acute lung injury therapy in the future.

## 1 Introduction

Acute lung injury (ALI) and acute respiratory distress syndrome (ARDS) arise from the disruption of the usual functioning of the alveolar epithelium. The injured alveoli show a strong inflammatory reaction with leukocyte accumulation, activation of coagulation within the alveolar spaces and small blood vessels, and injury to the lining of both air and blood pathways.^1^ Additionally, lung endothelium damage increases proinflammatory factor production and adhesion molecule expression, which promotes leukocyte attachment and passage into the lung, causing further harm through the release of enzymes, cytokines, reactive oxygen species (ROS), and active lipids.^1^ Treatment strategies that target the repair of the alveolar epithelium are essential in tackling ARDS’s high mortality. Data from the LUNG-SAFE (Large Observational Study to Understand the Global Impact of Severe Acute Respiratory Failure) study, which examined 29 144 ICU patients in 50 countries in the 2014 winter, showed a 10% incidence of ARDS in ICU patients and a 23% occurrence in those on mechanical ventilation. The study also found mortality rates of 34.9% for mild ARDS, 40% for moderate, and 46.1% for severe ARDS cases.^2^ On the other hand, coronavirus disease 2019 (COVID-19) was caused by severe acute respiratory syndrome coronavirus 2 (SARS-CoV-2). A global literature survey reported the development of COVID-19-associated ARDS in hospitalized patients from the beginning of the COVID-19 pandemic in January until the end of July 2020. Among hospitalized COVID-19 patients, approximately 1/3 (33%) develop ARDS, 1/4 (26%) require transfer to an ICU, 1/6 (16%) receive invasive mechanical ventilation (IMV), and 1/6 (16%) die. For COVID-19 patients transferred to an ICU, nearly 2/3 (63%) receive IMV and 3/4 (75%) have ARDS.^3^

Numerous treatment approaches have been explored to address inflammation and its effects in the context of ALI/ARDS. Current anti-inflammatory strategies such as corticosteroids, neutrophil elastase inhibitors, GM-CSF, statins, ω3 fatty acid, or therapies targeted at improving lung mechanics such as surfactants, inhaled β agonists, and NO have not demonstrated a reduction in mortality. The only treatments that have shown improved survival are supportive measures that minimize pressure-induced lung injury during mechanical ventilation including lung-protective ventilation with neuromuscular blockers and prone positioning. These supportive treatments continue to be the primary focus of ARDS care.^4^

Within 72 h of the onset of lung injury, there is a notable infiltration of macrophages and neutrophils in lung tissue. This process leads to the destruction of type I alveolar epithelial cells, increasing the microvascular permeability in the lungs. Consequently, acute pulmonary edema occurs, filling the alveoli with a substantial amount of fluid and exudate, resulting in severe hypoxia and potentially fatal outcomes. Collagen levels rise significantly, leading to the thickening of the alveolar wall interstitium and fibrosis. In response to infection or trauma, proinflammatory factors like tumor necrosis factor-α (TNF-α), interleukin-1 (IL-1), interleukin-6 (IL-6), and interleukin-8 (IL-8) become upregulated, initiating and sustaining inflammation during lung damage. These activated inflammatory cells produce O^2•–^ (main ROS), which is rapidly converted to H_2_O_2_ by superoxide dismutase. The production of chemokines, cytokines, and proinflammatory mediators in lipopolysaccharide (LPS)-induced ALI is largely regulated by the nuclear factor kappa-light-chain-enhancer of activated B cells (NF-κB) pathway and its upstream components, notably mitogen-activated protein kinases (MAPKs), with a particular emphasis on p38 MAPK and c-Jun N-terminal kinases (JNK). NF-κB is activated by LPS, prompting the translocation of NF-κB dimers (p65/p50 subunits) to the nucleus following degradation.^1,5^ Consequently, the inhibition of inflammatory mediators may hold therapeutic potential for ALI/ARDS.^6^

Fingolimod, also known as FTY720, is an immunosuppressant initially approved as a first-line treatment for relapsing types of multiple sclerosis.^7^ More recently, it was discovered that FTY720 can help maintain the integrity of endothelial cells by activating S1P1 receptors. This action improves endothelial barrier function, reducing vascular permeability, alveolar bleeding, and immune cell infiltration.^8^ Animal study also demonstrated that FTY720 reduced systemic and local tissue levels of IL-6 resulting in down-regulation of S1PR1 implicating S1P signaling via S1PR1 as underlying elevated IL-6.^9^ In a different context, nobiletin (NOB), a dietary polymethoxylated flavonoid found in citrus fruits, has demonstrated its potential by reducing intracellular ROS generation and inhibiting the release of inflammatory cytokines, including TNF-α and interferon-γ by macrophages in an animal model of liver injury.^10^ NOB significantly decreased the levels of inflammatory cytokines and blocked the phosphorylation of NF-κB p65 and IκBα induced by LPS.

Since both drugs carried anti-inflammation effects, many studies have applied FTY720 or NOB in ALI; in a rat model of LPS-induced ALI, a single intraperitoneal injection of FTY720 effectively inhibited NF-κB activation, reduced inflammatory cytokine levels (TNF-α, IL-6, and IL-1β), and decreased the infiltration of inflammatory cells. FTY720 also alleviated lung tissue injury, reducing pulmonary edema and improving arterial oxygen levels and overall condition in ALI-affected rats. These results indicate that FTY720 has a protective effect against LPS-induced ALI, likely via the inhibition of NF-κB activation and the regulation of inflammatory pathways to ameliorate alveolar capillary barrier dysfunction.^11^ Another study investigated the anti-inflammatory effects of NOB in LPS-stimulated A549 cells and LPS-induced ALI in mice. The animals were given NOB or dexamethasone as a pretreatment before LPS exposure. Results showed that NOB significantly reduced lung damage, decreased inflammation via reducing TNF-α, IL-6, and NO in BALF, and inhibited the activation of NF-κB in both cell and animal models. This suggests that NOB has a protective effect against ALI, possibly by inhibiting NF-κB activation and the resulting inflammatory response.^12^

The integration of small-molecule drugs in combination therapies is a well-established clinical approach. Pioneering the field, the encapsulation of these drugs within single molecular carriers holds the promise of finely tuned and targeted drug release. However, some key issues should be considered especially when these drugs carried toxicity. Frequent adverse effects include fatigue, gastrointestinal disturbance, headache and upper respiratory tract infection and more serious, atrioventricular block, symptomatic bradycardia, herpetic viral infections and macular edema and were noted at FTY720 oral use.^13^ Another issue is biopharmaceutical limitations, such as solubility and oral bioavailability. Despite promising preclinical results, NOB faces significant biopharmaceutical challenges, including low solubility in water (below 1 μg/mL), limited ability to cross biological barriers, and low bioavailability.^14^ When compared to conventional medications, nanodrug formulations have physical and biological advantages such as improved solubility and pharmacokinetics, higher efficacy, and less toxicity.^15,16^ Our study aims to solve the problem of low drug bioavailability and prolong the sustained release time. Combination therapy of synergistic effects can reduce the side effects of drugs by reducing the drug dose.

While oral administration is the most common and patient-friendly method of drug delivery, it poses challenges when dealing with drugs that have limited solubility, short sustainability, and low oral absorption rates. Achieving the necessary therapeutic concentration for such drugs can be particularly challenging.^17^ For optimal airway deposition, the use of biodegradable nanoparticles in inhalation platforms presents an enticing solution. This approach not only ensures the safeguarding of drug payloads against enzymatic or hydrolytic degradation but also minimizes inactivation, thereby reducing the risk of local and systemic side effects. Additionally, it allows for a more efficient reduction in total dose, dosing frequency, and overall systemic exposure.^18,19^

**Figure 1:**
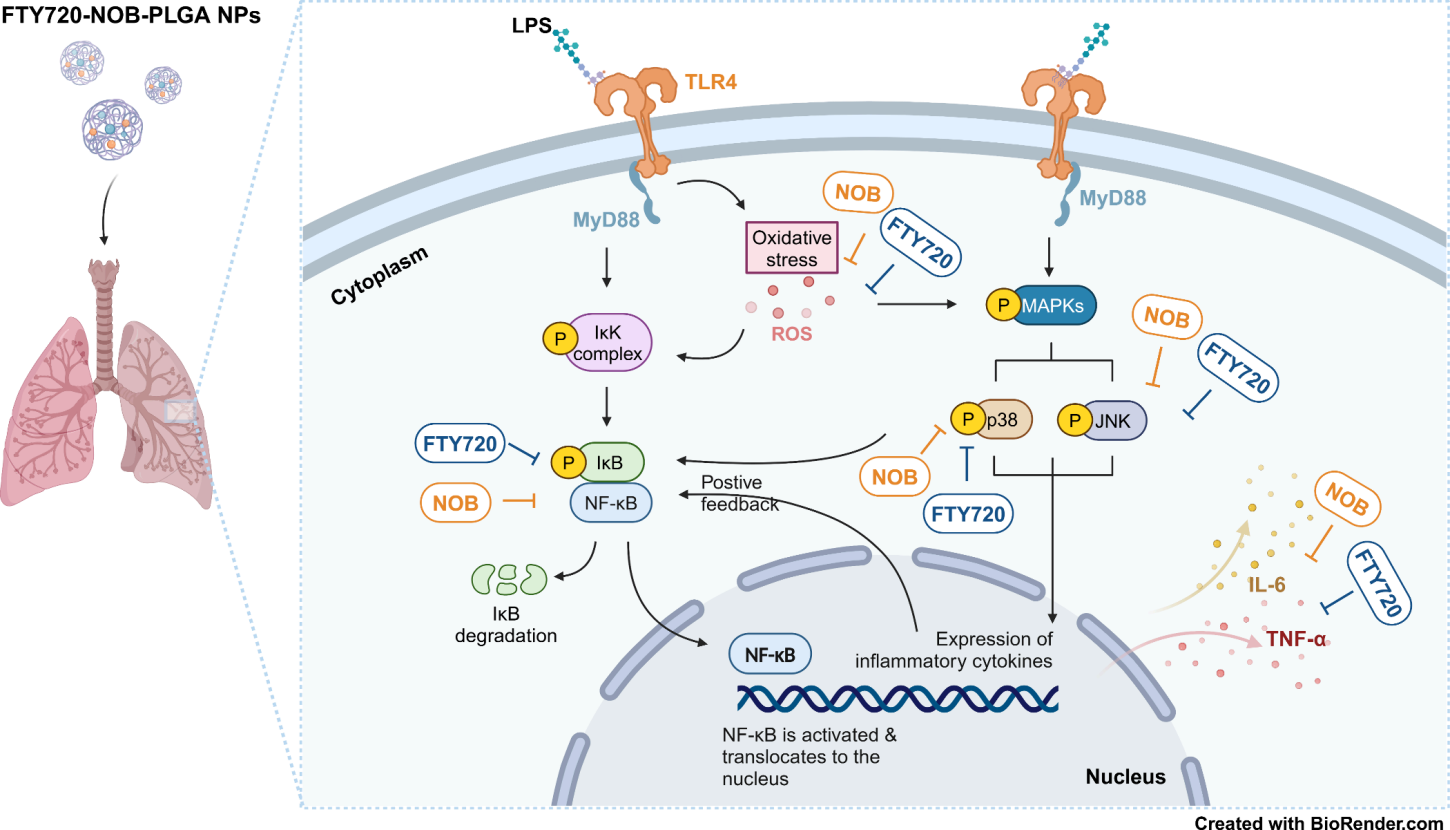
Scheme of encapsulating the synergistic effect of the dual drugs in the treatment of ALI.

In the current study, we develop an inhalation platform with nanodrug formulations of FTY 720 and NOB. We used poly(lactic-*co*-glycolic) acid (PLGA) to encapsulate FTY720 and NOB in PLGA (FTY720-NOB-PLGA NPs) and make sure of the good dispersion, adequate size, and good stability of the nanoformulation. In normal lung fibroblast cell, we confirmed the safety *in vitro*. We further verify the anti-inflammation of FTY720-NOB-PLGA NPs *in vitro* and *in vivo* via inhibiting IL-6 and TNF-α secretion, ROS production, and p38 MAPK activation (Figure 1). Our inhalation system, equipped with an inhalation tower, provides precise environmental control and respiratory parameter monitoring during LPS exposure and treatment.

## 2 Results

### 2.1 Characterization of FTY720-NOB-PLGA NPs

**Figure 2:**
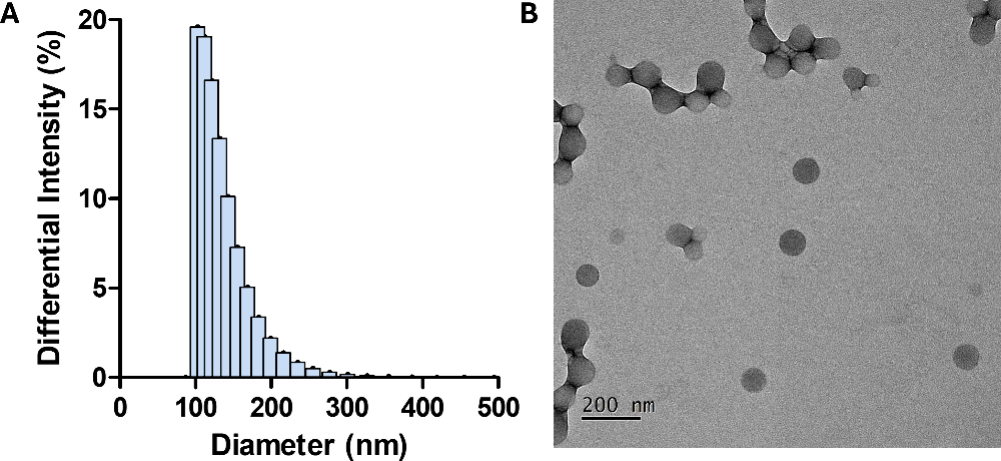
(A) The number distribution of coencapsulated FTY720 and NOB NPs was prepared by the emulsification method using PLGA. (B) The morphology of the FTY720-NOB-PLGA NPs with a field of view of 30K.

The hydrophilic drug FTY720 and the hydrophobic drug NOB were both encapsulated in the PLGA polymer. The size distribution of the resultant NPs was approximately 126 ± 30.18 nm, as determined by dynamic light scattering (DLS) (Figure 2A). Supplementary Table 1 disclosed that the polydispersity index (PI) was 0.213, and the ζ potential of the FTY720-NOB-PLGA NPs was −13.55 ± 2.42 mV. An image from transmission electron microscopy of FTY720-NOB-PLGA NPs can be seen in Figure 2B.

**Figure 3:**
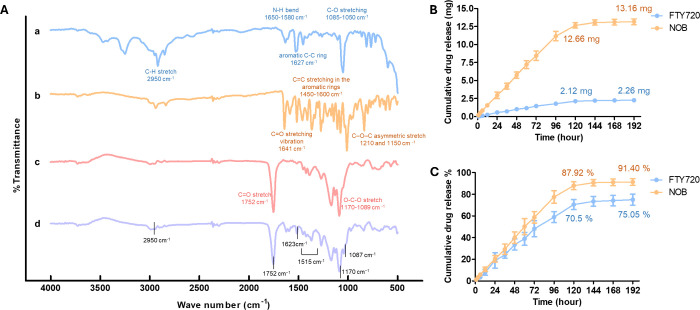
(A) FTIR spectra of the (a) FTY720, (b) NOB, (c) PLGA, and (d) FTY720-NOB-PLGA NPs with wave numbers from 500 to 4000 cm^–1^. The FTY720-NOB-PLGA NPs underwent drug release at 1× PBST and 37 °C. Concentrations of FTY720 and NOB were determined at wavelengths of 200 and 330 nm and expressed as (B) grams of cumulative drug and (C) percent of total cumulative drug.

The Fourier transform infrared spectroscopy (FTIR) spectra are shown in Figure 3A. The functional group of FTY720 was confirmed by the peak at 2950 cm^–1^ related to the C–H stretching range, and the N–H bending (primary amines) was located at 1650–1580 cm^–1^, the aromatic C–C ring was at 1627 cm^–1^, and the very strong sharp band at 1085–1050 cm^–1^ corresponded to the C–O stretching in primary alcohols. The C=O stretching vibration was found at 1641 cm^–1^, the aromatic C=C ring at 1450–1600 cm^–1^, and the C–O–C asymmetric stretching at 1210 and 1150 cm^–1^, indicating that the functional group belongs to NOB. The carbonyl group stretching in PLGA was obtained at 1752 cm^–1^, and the C–O group stretching appeared at 1089–1170 cm^–1^. The FTIR spectra of the FTY720-NOB-PLGA NPs showed characteristic bands at 2950, 1623, and 1087 cm^–1^, which could be attributed to the C–H stretching, the N–H bending, aromatic C–C ring, and the C–O stretching in primary alcohols, for FTY720. The functional groups at 1515 cm^–1^ corresponded to the aromatic C=C ring of NOB, and 1752 and 1170 cm^–1^ to the carbonyl group stretching and the C–O group of PLGA, respectively. According to the above characterization, we successfully encapsulated FTY720 and NOB by using PLGA polymer.

### 2.2 Drug Release Assay of FTY720-NOB-PLGA NPs and NOB-PLGA NPs

After measurement, each synthesis of FTY720-NOB-PLGA NPs encapsulates an average of 2.89 mg of FTY720 and 14.93 mg of NOB. The drug encapsulation rates of FTY720 and NOB were 57.87 and 74.68%, respectively, and drug loading efficacy was 2.89 and 14.93%, respectively (Supplementary Table 2 and Table 3). To evaluate the sustained drug release of FTY720-NOB-PLGA NPs, the *in vitro* release kinetics of FTY720 and NOB from PLGA NPs were investigated in phosphate-buffered saline with Tween-20 PBST (pH 7.2) at 37 °C. The result is shown in Figure 3B,C.

The release was almost 10% within 12 h, while the PLGA NPs displayed a slow release from FTY720 and NOB after 72 h. The drug release of FTY720-NOB-PLGA NPs reached saturation at 144 h (5 days). Finally, 75.05% (2.26 mg) and 91.4% (13.16 mg) of FTY720 and NOB were released at 192 h (8 days). The results indicated that the FTY720-NOB-PLGA NPs were capable of sustained release and were adequately stable. We also assessed the sustained drug release characteristics of NOB-PLGA NPs, and the outcome is presented in Supplementary Figure 1. The results indicated that over 40% of NOB was released within the initial 12 h, pointing to a rapid release profile without prolonged sustained effects. The drug release reached saturation at 96 h, with 77.05% (12.22 mg) of NOB ultimately released. The findings imply that the sustained drug release behavior of FTY720-NOB-PLGA NPs is superior to that of the NOB-PLGA NPs.

### 2.3 The Safety of FTY720-NOB-PLGA NPs to Normal Cells and the Anti-Inflammation Effect to Macrophage

**Figure 4:**
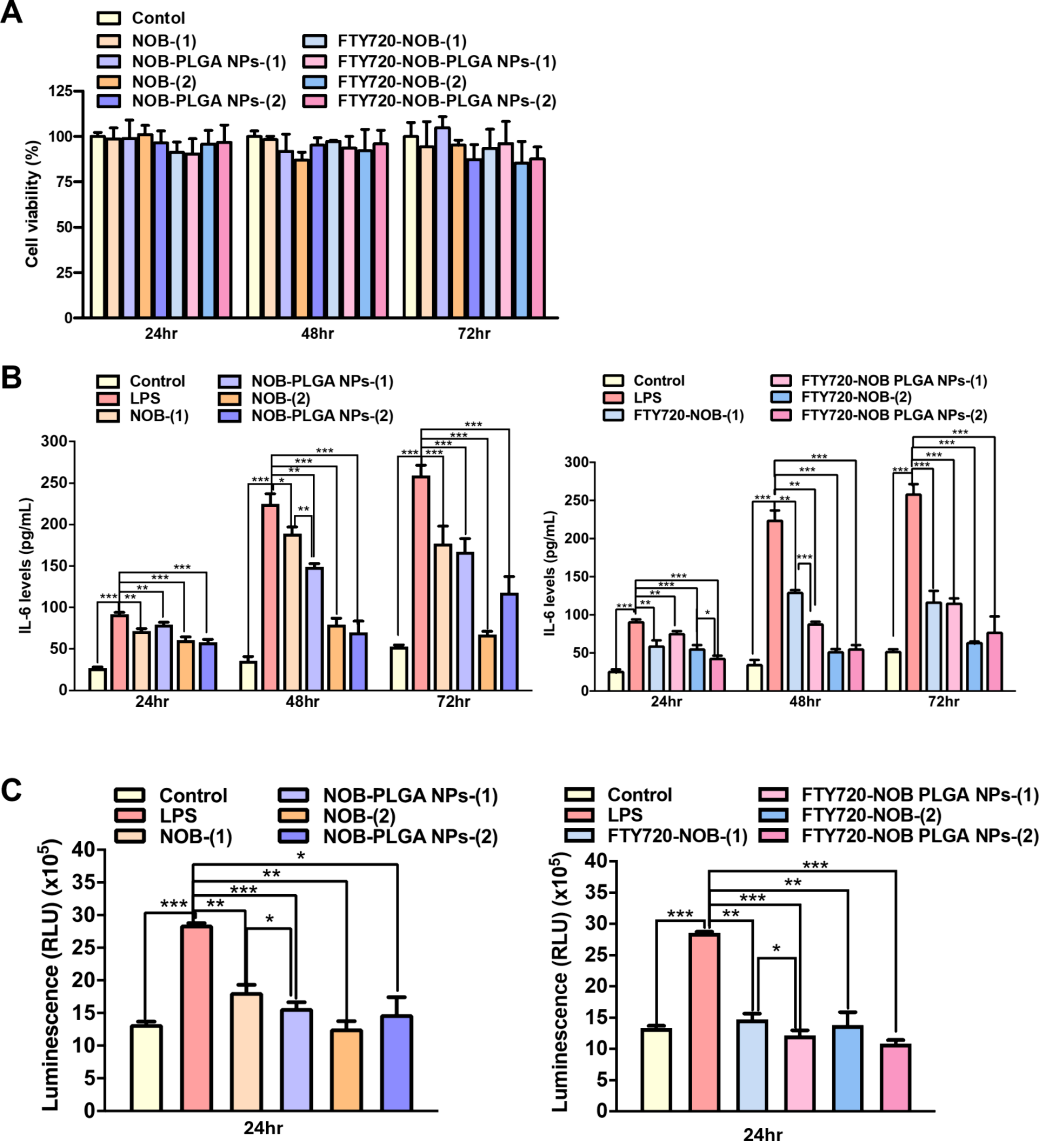
(A) Normal lung fibroblasts (IMR-90 cells) were treated with pure drug (NOB-(1) 2.5 × 10^–3^ mg/mL and NOB-(2) 5 × 10^–3^ mg/mL), drug combination (FTY20-NOB-(1) 4.06 × 10^–4^ mg/mL FTY20 + 2.5 × 10^–3^ mg/mL NOB and FTY20-NOB-(2) 8.125 × 10^–4^ mg/mL FTY20 + 5 × 10^–3^ mg/mL NOB) and PLGA encapsulated NPs (NOB-PLGA-NPs, FTY-NOB-PLGA). CCK-8 assay was used for measurement of viability. After stimulation with LPS for 24 h, Raw264.7 cells were treated with pure drug (NOB-(1) 5 × 10^–3^ mg/mL and NOB-(2) 1 × 10^–2^ mg/mL), drug combination (FTY20-NOB-(1) 8.125 × 10^–4^ mg/mL FTY20 + 5 × 10^–3^ mg/mL NOB and FTY20-NOB-(2) 1.625 × 10^–3^ mg/mL FTY20 + 10^–2^ mg/mL NOB) and PLGA encapsulated NPs (NOB-PLGA-NPs, FTY-NOB-PLGA NPs) to investigate their impact on secretion of IL-6 (B) and ROS (C) using ELISA assay and Ultra-Glo recombinant luciferase and d-cysteine assay, respectively. The values presented are the mean ± SD. *p* values were obtained using the Student’s *t* test (***p < 0.001, **p < 0.01 and *p < 0.05).

We initially assessed the *in vitro* cytotoxicity of free drugs and PLGA-encapsulated nanoparticles on normal lung fibroblast (IMR-90) cells using the CCK-8 assay. As shown in Figure 4A, exposure to varying combinations of free FTY720 (4.625 or 0.8123 × 10^–4^ mg/mL) and NOB (2.5 or 5 × 10^–3^) displayed no significant difference in cytotoxicity compared to the control group. While slightly increased toxicity was observed at higher doses of free drugs (Supplementary Figure 2), cell viability remained above 60% with PLGA-encapsulated nanoparticles.

In ALI/ARDS, macrophages play a pivotal role in lung tissue damage by producing cytokines, such as IL-6, which enhance alveolocapillary permeability and recruit neutrophils to the alveolar and epithelial spaces.^20^ Macrophages also generate ROS, leading to injury in endothelial and alveolar epithelial cells. Initially, we confirmed LPS-induced IL-6 secretion in RAW 264.7 cells (monocyte/macrophage-like cells) in a dose- and time-dependent manner (Supplementary Figure 3). In Figure 4B, we successfully demonstrated that both NOB-PLGA and NOB treatments for 24 h could inhibit IL-6 secretion after 24, 48, and 72 h of LPS exposure. Furthermore, we employed the Ultra-Glo recombinant luciferase and d-cysteine assay to investigate the ability of PLGA-encapsulated PLGA to inhibit ROS production in RAW 264.7 cells after 24 h of LPS exposure. As anticipated, both NOB and NOB-PLGA effectively inhibited ROS production (Figure 4C), and FTY720-NOB-PLGA NPs also exhibited ROS-inhibitory effects, like the dual drug treatment.

### 2.4 The Anti-Inflammation Effect in Mice of LPS-Induced ALI

#### 2.4.1 LPS-Induced ALI Animal Model and Inhalation Tower System

Finally, we assessed the anti-inflammatory activity of FTY720-NOB-PLGA NPs in an LPS-induced ALI animal model. Unanesthetized mice were placed in a nose-only inhalation tower system (DSI Buxco respiratory solutions), with animals gently restrained by a neck clip to prevent thoracic compression. This setup allowed for open airways and spontaneous breathing through the nose, as depicted in Supplementary Figure 4A. Following a 1 h exposure to LPS, the unanesthetized mice were further subjected to aerosolized solutions, including PBS, NOB-PLGA NPs, and FTY720-NOB-PLGA NPs, for a duration of 30 min. The aerosolization process was meticulously controlled in terms of pressure, temperature, and humidity conditions, as illustrated in Supplementary Figure 4B,C. Throughout the aerosol exposure, we recorded various parameters of pulmonary function using plethysmography, as represented in Supplementary Figure 4D. To evaluate the distribution of FTY720-NOB-PLGA NPs within the respiratory alveoli following administration through an inhalation tower system, lung biodistribution patterns postinhalation of PLGA-Cy5 nanoparticles were documented utilizing confocal microscopy. As indicated in Supplementary Figure 5A, a uniform distribution of Cy5 fluorescence with high intensity was observed, suggesting the effective delivery of PLGA-Cy5 to the alveoli via the inhalation system. Furthermore, cellular uptake and internalization of the nanoparticles were examined at an elevated optical magnification (Supplementary Figure 5B, 100×) in the confocal microscope. The white arrow indicated that the drug in encapsulated form was indeed delivered to the lung alveoli.

#### 2.4.2 The Impact of Inhalation of PLGA Nanoparticle on IL-6 and TNF-α Secretion and Lung Alveolar Structure, and NF-κB Expression in LPS-Induced ALI Animal Model

**Figure 5:**
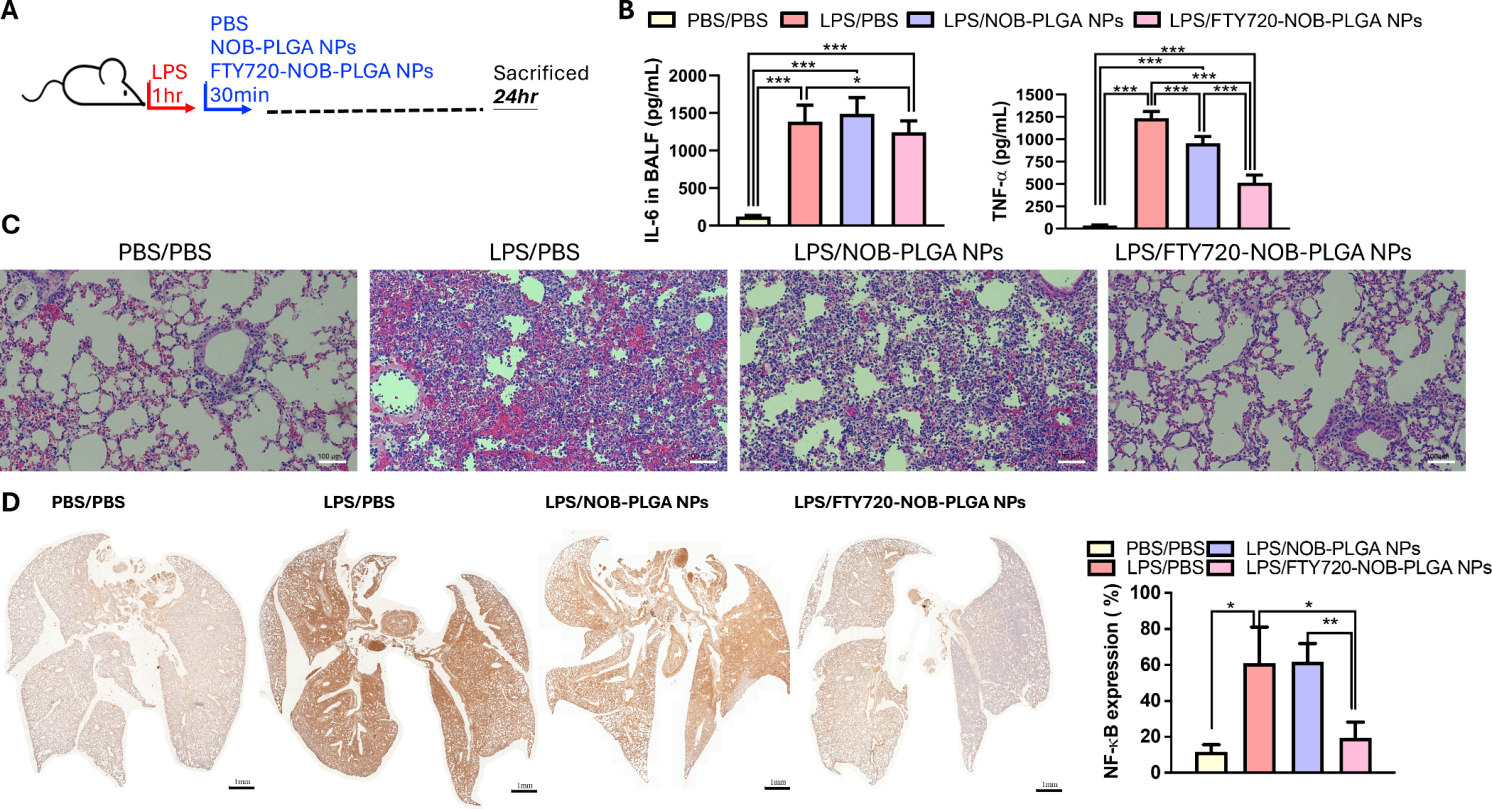
(A) Schematic diagram illustrating the design of the animal model. (B) Evaluation of IL-6 and TNF-α secretion in bronchoalveolar lavage fluid (BALF) from LPS-induced lung injury mice (*n* = 3 per group) after 24 h of treatment with PBS, NOB-PLGA NPs, and FTY720-NOB-PLGA NPs using an ELISA kit. (C) Histopathological examination of lung tissues in mice (*n* = 3 per group) with LPS-induced lung injury treated with PBS, NOB-PLGA NPs, and FTY720-NOB-PLGA NPs at 24 h, displayed at an original magnification of 100×. (D) Panoramic images of NF-κB obtained through the TissueFAXs platform and quantification of NF-κB expression percentage in the entire lung section (***p < 0.001, **p < 0.01, and *p < 0.05).

Since the release of IL-6 and TNF-α in bronchoalveolar lavage fluid (BALF) contributes to increased alveolocapillary permeability and the recruitment of neutrophils, our initial investigation aimed to determine whether *in vivo* inhibition of IL-6 and TNF-α secretion could be achieved through PLGA-encapsulated NPs inhalation for 30 min (as depicted in Figure 5A). The results were enlightening, as IL-6 and TNF-α levels in BALF were significantly higher in the PBS (LPS/PBS) and NOB-PLGA (LPS/NOB-PLGA NPs) groups but notably lower in the FTY720-NOB-PLGA (LPS/FTY720-NOB-PLGA NPs) group. This highlights the effectiveness of FTY720-NOB-PLGA NPs inhalation in reducing the release of IL-6 and TNF-α 24 h after the onset of lung injury (Figure 5B). Conversely, NOB-PLGA NPs inhalation had no significant impact on cytokine release in the animal model. Histological examination using hematoxylin and eosin (H&E) staining unveiled typical lung injury findings in the PBS group, characterized by extensive alveolar hemorrhages, widespread accumulation of inflammatory cells in the alveolar cavity, and diffuse alveolar wall thickening. In comparison, inhalation treatment with NOB-PLGA NPs led to reduced alveolar hemorrhage and inflammatory cell infiltration but still showed signs of diffuse alveolar wall thickness. Intriguingly, the FTY720-NOB-PLGA NPs groups displayed localized alveolar thickness and rarely identified alveolar hemorrhage and inflammatory cell infiltration, reinforcing the protective effect of FTY720-NOB-PLGA NPs inhalation in the animal model (Figure 5C). Immunohistochemistry (IHC) staining showed that the expression area of NF-κB in the FTY720-NOB-PLGA NPs group remained smaller than that in the PBS treatment group (LPS/PBS). Conversely, NOB-PLGA NPs inhalation had no significant impact on the expression of NF-κB in the animal model (Figure 5D).

**Figure 6:**
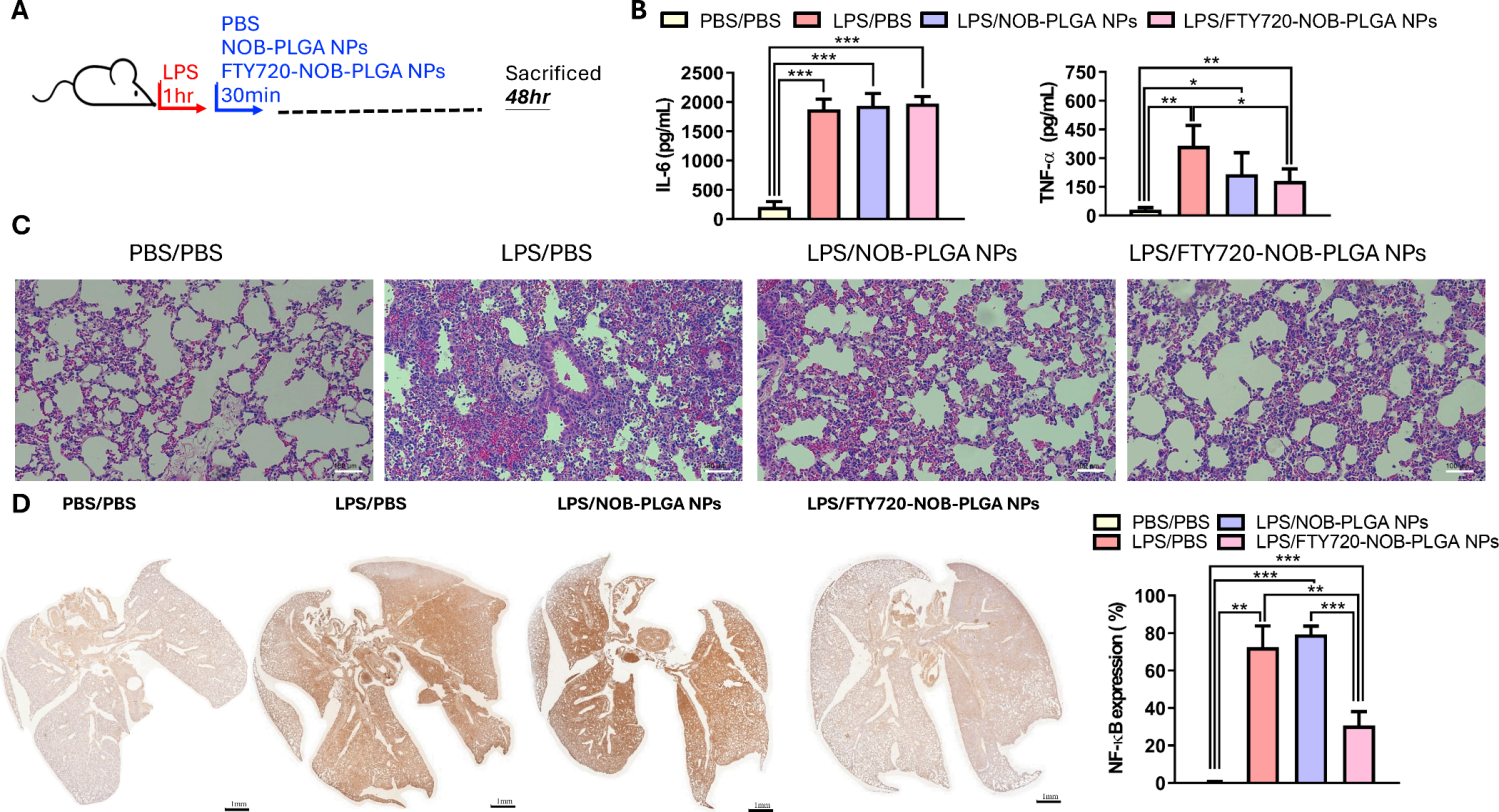
(A) Schematic diagram of the animal model examining the endurance of FTY720-NOB-PLGA NPs’ efficacy over a 48 h period. (B) Assessment of IL-6 and TNF-α secretion in bronchoalveolar lavage fluid (BALF) from LPS-induced lung injury mice (*n* = 3 per group) 48 h post-treatment with PBS, NOB-PLGA NPs, and FTY720-NOB-PLGA NPs using an ELISA kit. (C) Lung pathology evaluation in mice (*n* = 3 per group) with LPS-induced lung injury treated with different drugs at 48 h, displayed at an original magnification of 100×. (D) Panoramic images of NF-κB obtained through the TissueFAXs platform, along with quantification of NF-κB expression percentage in the entire lung section (***p < 0.001, **p < 0.01, and *p < 0.05).

We proceeded to examine the endurance of FTY720-NOB-PLGA NPs’ efficacy over a 48 h period (as shown in Figure 6A). After inducing lung injury with LPS for 48 h, inhaled FTY720-NOB-PLGA NPs were able to effectively reduce the release of TNF-α, though they did not impact IL-6 secretion in bronchoalveolar lavage fluid. In contrast, the NOB-PLGA NPs treatment failed to inhibit the secretion of both TNF-α and IL-6 (Figure 6B). Histological analysis unveiled persistent alveolar hemorrhage and inflammatory cell infiltration in the PBS/LPS group. In the NOB-PLGA NPs group, evident loss of alveolar architecture, increased interstitial thickness, and the presence of immune cells and red blood cell infiltration were observed. Though the FTY720-NOB-PLGA NPs group displayed a notable absence of alveolar hemorrhage and inflammatory cell infiltration, the alveolar thickness was more pronounced than in the control group (Figure 6C). IHC staining showed that the expression area of NF-κB in the FTY720-NOB-PLGA NPs group remained smaller than that in the PBS treatment group (LPS/PBS), although it was larger than that in the normal control (PBS/PBS) group (Figure 6D).

**Figure 7:**
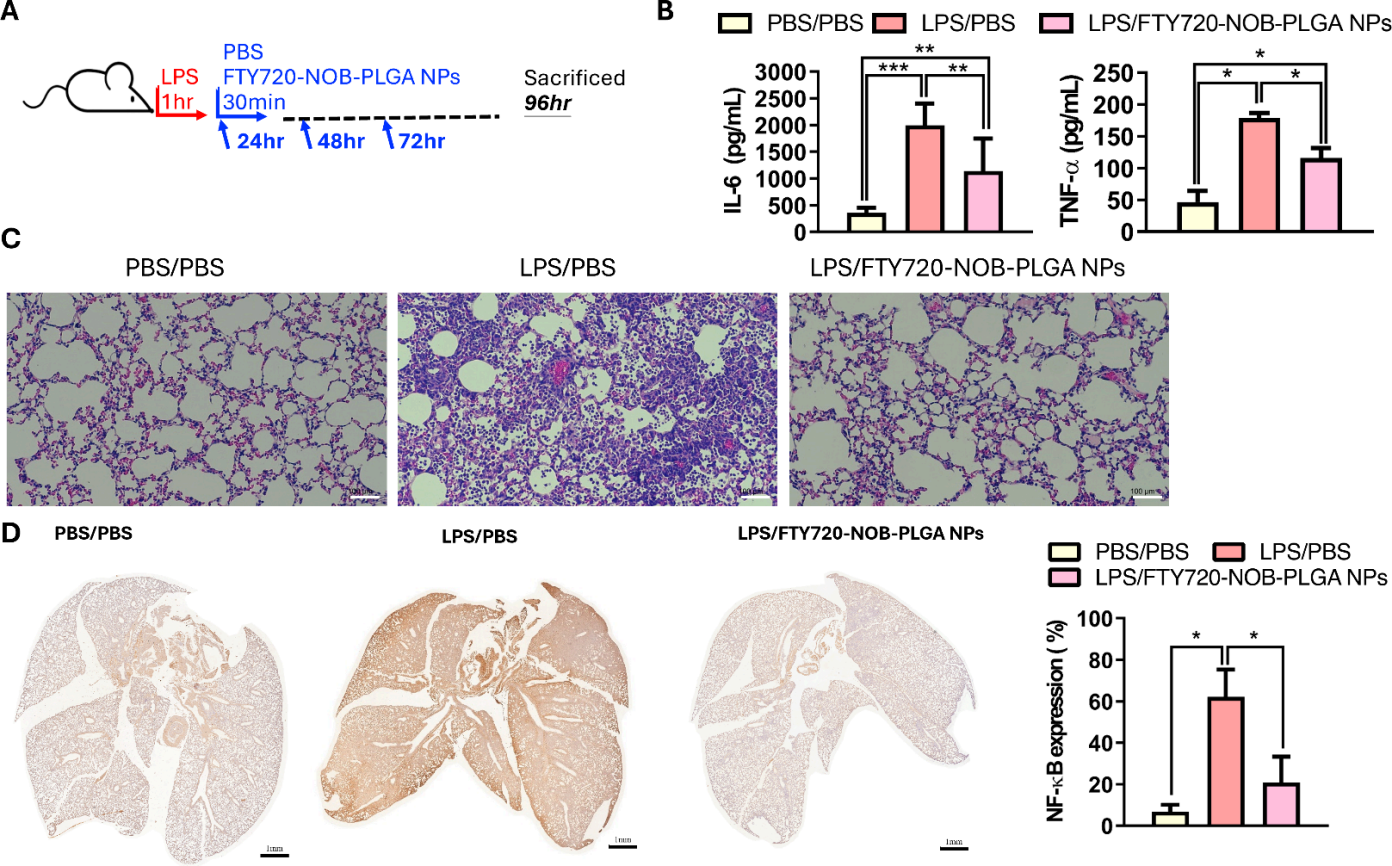
(A) Schematic illustration of FTY720-NOB-PLGA NPs inhalation for 30 min daily over three consecutive days, followed by sacrifice at 96 h. (B) Comparison of the impact of different treatments (PBS and FTY720-NOB-PLGA NPs; 30 min/day for 3 days) on IL-6 and TNF-α secretion in BALF using an ELISA kit. (C) Lung pathology assessment in mice (*n* = 3 per group) with LPS-induced lung injury treated with different drugs for three consecutive days. (D) Panoramic NF-κB images obtained through the TissueFAXs platform, and quantification of NF-κB expression percentage in the entire lung section. Mean ± SD values are presented, and *p*-values were determined using the Student’s *t* test (***p < 0.001, **p < 0.01, and *p < 0.05).

In the pursuit of maximizing the benefits of inhaled FTY720-NOB-PLGA NPs treatment (as depicted in Figure 7A), we altered our treatment strategy. Mice were subjected to inhaled FTY720-NOB-PLGA NPs for 30 min daily over the course of 3 days, after which they were sacrificed at 96 h. We found the FTY720-NOB-PLGA NPs inhalation can effectively reduce the release of TNF-α as well as IL-6 secretion in BALF (Figure 7B). Histological findings revealed that the alveolar structure in the FTY720-NOB-PLGA NPs group closely resembled that of the control group (Figure 7C), in stark contrast to that of the LPS-induced group. Moreover, the expression area of NF-κB in the FTY720-NOB-PLGA NPs group was notably lower than that in the LPS-induced group, as evident in Figure 7D. Notably, there was no statistical difference in NF-κB expression between the FTY720-NOB-PLGA NPs group and the normal control group, highlighting the efficacy of this innovative treatment approach.

**Figure 8:**
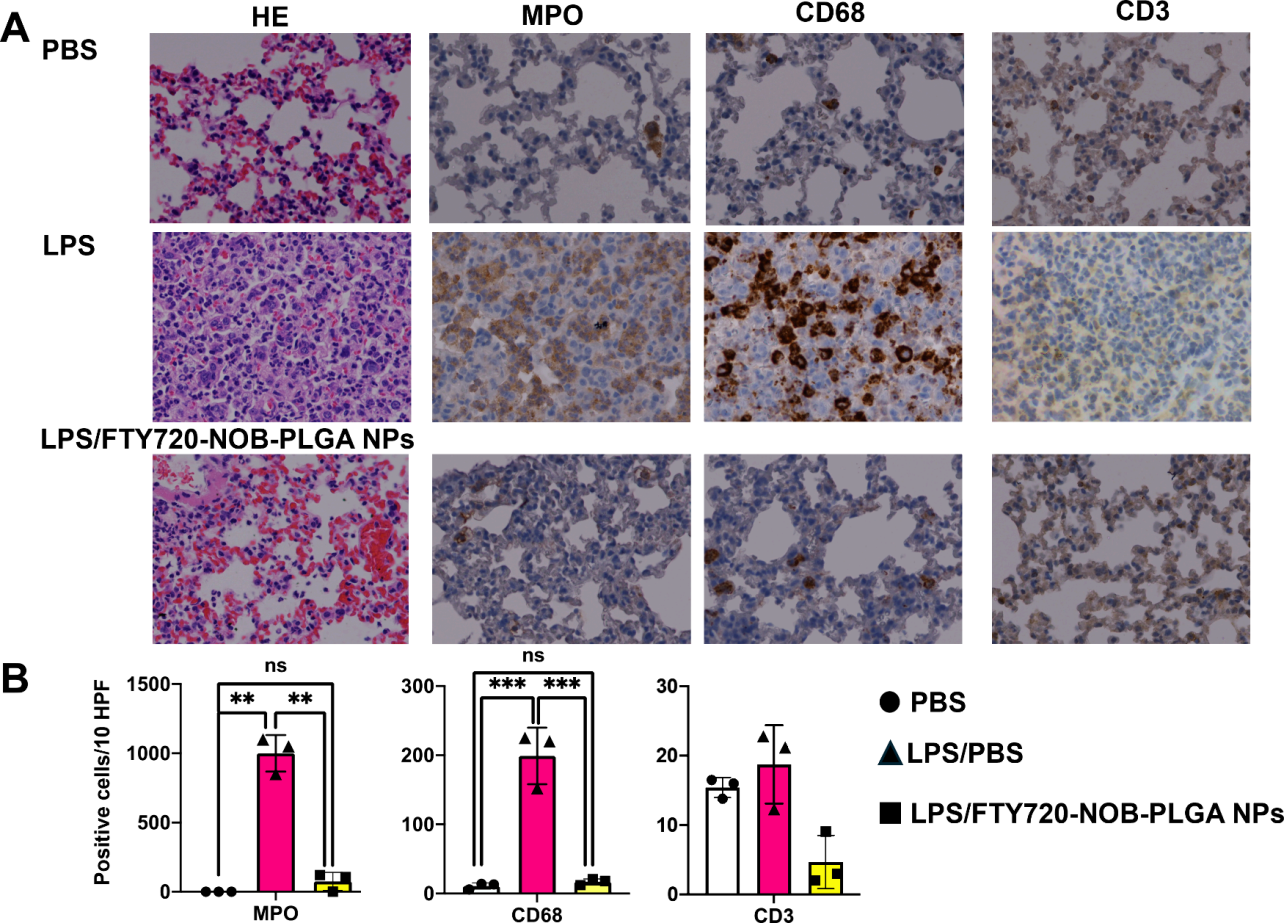
Histological analysis of lung tissue sections from control, LPS-induced acute lung injury, and post-treatment with FTY720-NOB-PLGA NPs. (A) Tissue sections were stained using H&E and immunohistochemical staining for MPO, CD68, and CD3. Positive cells are brown (magnification, 400×). Scale bar, 10 μm. (B) Quantification of neutrophils (MPO), macrophages (CD68), and T cells (CD3) per high-power field (HPF) derived from IHC data (*n* = 3 per group). Data are presented as mean ± SD. Statistical significance was assessed using the Student’s *t* test (***p < 0.001, **p < 0.01).

**Figure 9:**
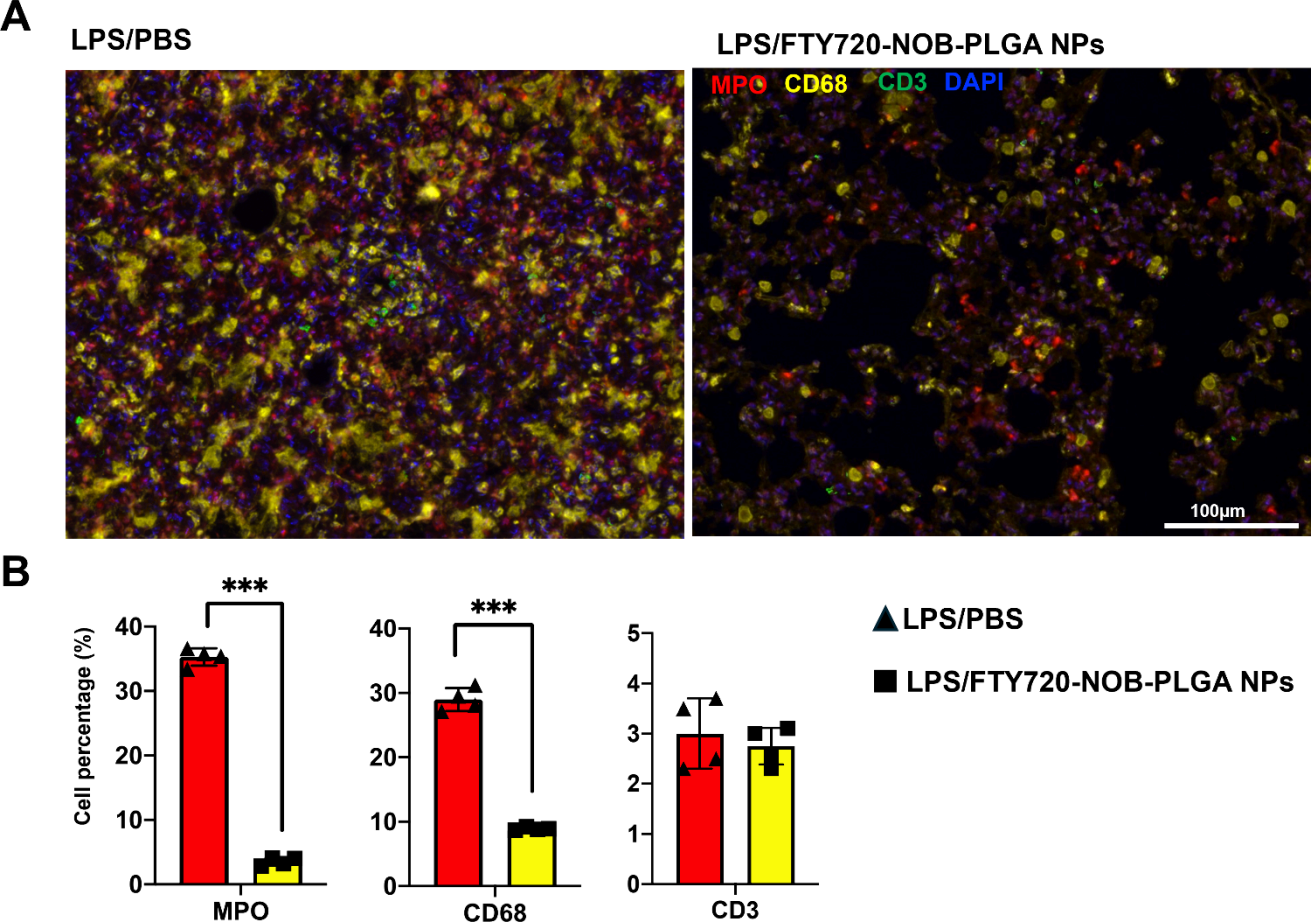
Multiplexed mIF assay of immune cell infiltration. (A) Representative images from the mIF assay on lung tissue from mice post-LPS stimulation with or without FTY720-NOB-PLGA nanoparticle inhalation treatment, showing staining for MPO (neutrophils), CD68 (macrophages), CD3 (T cells), and DAPI (nuclei). Scale bar, 100 μm. (B) The bar graph illustrates the proportion of neutrophils, macrophages, and T cells infiltrating the lungs of mice following LPS stimulation with or without FTY720-NOB-PLGA nanoparticle inhalation treatment. Data are presented as mean ± SD, calculated from four fields per mouse lung. Statistical significance was determined using the Student’s *t* test (***p < 0.001).

To elucidate the internalization of FTY720-NOB-PLGA by immune cells, lung tissue after inhalation treatment underwent immunofluorescence staining. The results shown in Supplementary Figure 6 demonstrated the cellular uptake of FTY720-NOB-PLGA-Cy5 NPs in distinct immune cell populations, notably macrophages displaying F4/80 expression (Supplementary Figure 6A), neutrophils exhibiting myeloperoxidase expression (Supplementary Figure 6B), and T cells demonstrating CD3 expression (Supplementary Figure 6C). We further examined the recruitment of immune cells, specifically macrophages, neutrophils, and T cells, following LPS stimulation in the lung tissue of mice, as well as the changes in the abundance of these cells after inhalation treatment with FTY720-NOB-PLGA NPs. IHC staining revealed that LPS induced the recruitment of macrophages and neutrophils, which was effectively inhibited following inhalation treatment with FTY720-NOB-PLGA NPs (Figure 8A). As in the previous study,^21^ we observed no significant change in T cell recruitment following LPS stimulation. Additionally, the inhalation treatment with FTY720-NOB-PLGA nanoparticles did not affect T cell recruitment (Figure 8B). Using multiplex immunofluorescence (mIF), which enables the assessment of multiple markers within a single experiment,^22^ we compared the proportions of neutrophils, macrophages, and T cells infiltrating the lungs of mice following LPS stimulation with or without FTY720-NOB-PLGA NPs inhalation treatment. Consistent with the results from IHC, we observed a significantly higher proportion of macrophage and neutrophil infiltration in the lung tissue following LPS stimulation alone compared with LPS stimulation accompanied by FTY720-NOB-PLGA NPs inhalation treatment (Figure 9).

#### 2.4.3 The Impact of FTY720-NOS-PLGA NPs Inhalation Therapy on Organ Function

**Figure 10:**
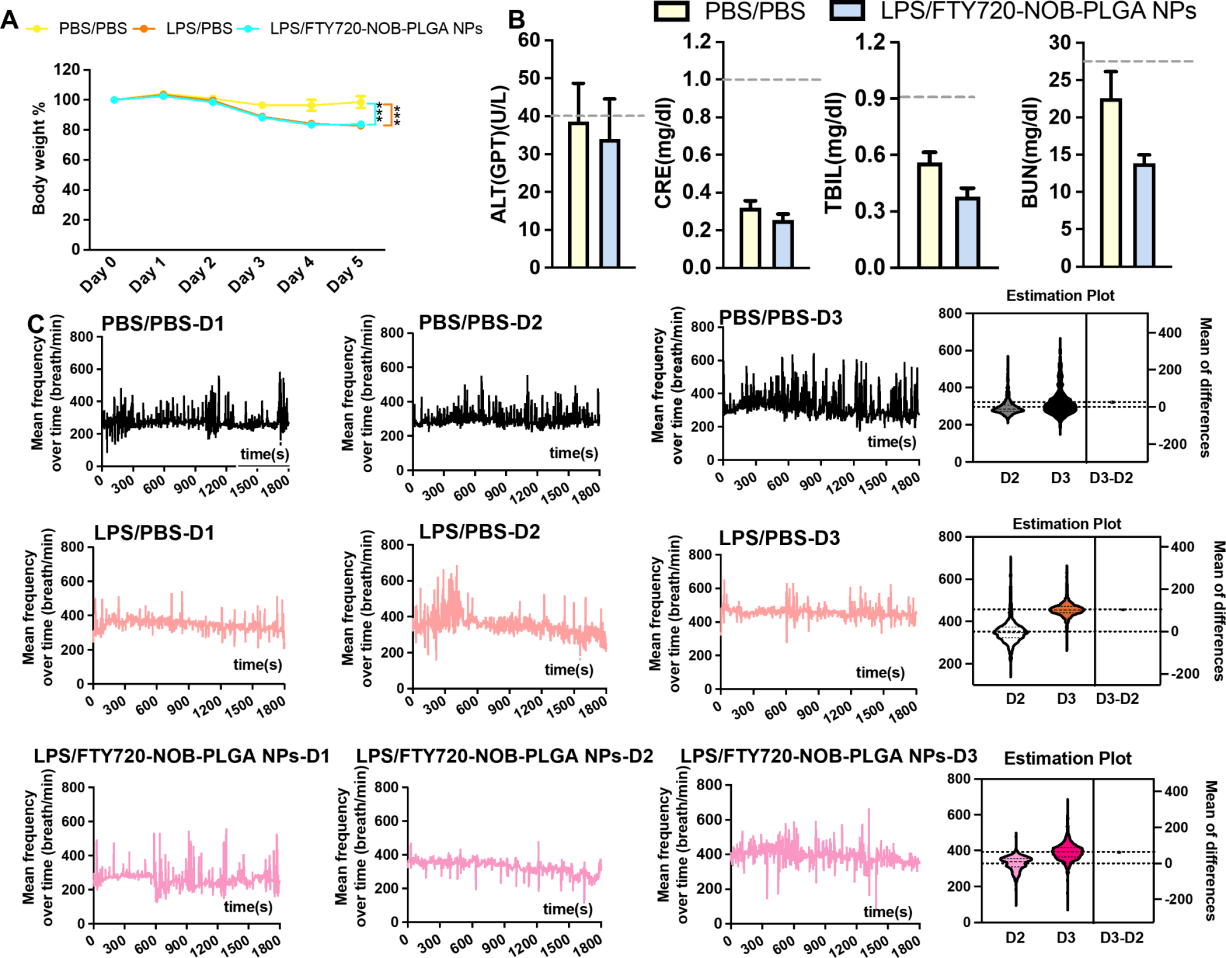
Body weight, organ function, and respiratory rate in control group and treatment group. (A) Body weight percentage of mice in response to LPS and FTY720-NOB-PLGA NPs. The values presented are the mean ± SD. *p* values were obtained using the Student’s *t* test (***p < 0.001). (B) Blood biochemical results of the FTY720-NOB-PLGA NPs following inhalation into mice (*n* = 5). The results show mean and standard deviation of alanine aminotransferase (ALT), creatinine (CRE), total bilirubin (TBIL), and blood urea nitrogen (BUN). (C) Respiratory parameters: Mean of breathing frequency was analyzed during day 1 to day 3 through plethysmography associated with the Allay restrainer.

Nanoparticles may be swiftly excreted through urine, potentially impacting renal function, while larger nanoparticles often get sequestered by liver and spleen cells, potentially following the hepatobiliary elimination pathway, which poses risks of liver and spleen toxicity.^23^ Therefore, we investigated whether inhaled PLGA nanoparticles induce organ dysfunction *in vivo*. Initially, LPS exposure resulted in approximately 20% body weight loss, but additional treatment with inhaled FTY720-NOB-PLGA NPs did not lead to further weight loss (Figure 10A). Moreover, liver enzyme and renal function levels in both the PBS group and the FTY720-NOB-PLGA NPs group remained within the normal range (Figure 10B). Through plethysmography, we observed an increase in mean respiratory frequency after LPS stimulation, especially in the PBS treatment group on day 3 (from 352.7 ± 58.4 breaths/min on day 2 to 457.2 ± 34.2 breaths/min on day 3). In contrast, although the mean respiratory frequency also increased in the FTY720-NOB-PLGA NPs group (from 329.5 ± 40.1 breaths/min on day 2 to 393.4 ± 49.3 breaths/min on day 3), the increase was less pronounced compared to that of the PBS treatment group (Figure 10C).

## 3 Discussion

In our current study, we are dedicated to substantiating the compelling synergistic effects of PLGA-encapsulated FTY720 and NOB in inhalation therapy. The strategic encapsulation of drug molecules within nanoparticles not only offers protection against enzymatic degradation but also capitalizes on well-established safety and efficacy. However, when it comes to addressing acute lung injury, the systemic safety of PLGA nanoparticles is a paramount concern. Factors such as hepatic first-passage metabolism, blood clearance, and kidney deposition need to be meticulously considered. Furthermore, the imperative for respiratory distress monitoring cannot be overstated, as certain drugs have the potential to induce lung damage or exacerbate existing injuries.^24^ Our study employed an innovative approach by utilizing inhaled FTY720-NOB-PLGA NPs, which have demonstrated effective reduction of LPS-induced lung injury through the inhibition of proinflammatory cytokines IL-6 and TNF-α secretion by immune cells. It is noteworthy that FTY720 can exhibit toxicity at high doses, potentially leading to more severe lung injuries. Additionally, NOB presents a challenge due to its poor water solubility. To address these limitations, we used biodegradable and biocompatible polymeric materials as carriers for concurrent drug delivery. The incorporation of various ratios of polyglycolic acid and polylactic acid results in diverse shapes, coating ratios, and release profiles.^25^

Inhalation drug delivery using nanoparticles necessitates careful consideration of particle size and charge.^26^ In this study, we utilized PLGA (50:50 copolymer composition) as the base material. Initially, 5 mg of FTY720 and 20 mg of NOB were encapsulated in PLGA. we synthesized different sizes and charges of PLGA NPs to select the optimum one for this study (Supplementary Table 4). FTY720 is more hydrophilic than NOB, which may result in less FTY720 being carried. If an excessive amount of FTY720 is added, there may be incomplete encapsulation, leading to the exposure of the amine group on the particle surface, causing a positive surface charge on the nanoparticles. By gradually reducing the amount of FTY720, we observed an increase in the encapsulation rate, nanoparticle size, and negative surface charge. Despite a slight increase in the PI value, the sample was generally considered acceptable for a highly polydisperse sample. According to the study by Kaminskas et al., who monitored the intravenous and pulmonary pharmacokinetics and pulmonary clearance of tritium-labeled (3H-propionate) PLGA nanoparticles of various sizes and charges in rats, they found that PLGA nanoparticles, regardless of size and charge, deposited uniformly in the upper and lower airways immediately after administration, with the majority usually localizing to deeper areas of the lung within 2 days.^27^ They also found that for the same size of PLGA nanoparticles with different charges, the surface positive charge nanoparticles had a clearance rate faster than that of the negative charge nanoparticles after pulmonary administration. This may be due to increased retention of cationic particles in the lungs through electrostatic interactions with the epithelium and mucous membranes leading to reduced mucociliary clearance.^28^ In a study evaluating PLGA particles of varying sizes loaded with paclitaxel (100–2500 nm), results indicated that 100 nm particles are more efficiently absorbed by lung epithelial cells and accumulate in the deeper lung areas, especially the alveoli.^29^ The optimum particle size for inhalation is 100–500 nm;^30^ in general, larger particles, ranging from 500 nm to 6 mm, are primarily cleared by phagocytosis.^31^ Therefore, this formulation (5 mg of FTY720 and 20 mg of NOB) was selected for the follow-up study due to its high drug carrying rate of both drugs, stability of the nanoparticles, and accumulation in the deeper lung areas after multiple administrations.

Figure 2 illustrates the characterization of FTY720-NOB-PLGA NPs. The DLS analysis revealed that the NPs had an approximate size of 126 ± 30.18 nm, with a PI numerical value of 0.213. A PI numerical value of 0.213 is generally considered acceptable for a highly polydisperse sample.^32^ The ζ potential results (Supplementary Table 1) indicated that FTY720-NOB-PLGA NPs carried a negative charge of −13.55 ± 2.42 mV. This negative charge was attributed to the presence of carboxyl groups in PLGA and the PVA surfactant on the outer layer of the FTY720-NOB-PLGA NPs. Additionally, the TEM images in Figure 2B demonstrate that the FTY720-NOB-PLGA NPs exhibit a spherical shape. Comparison of the FTIR spectra in Figure 3A, featuring (a) FTY720, (b) NOB, (c) PLGA, and (d) FTY720-NOB-PLGA NPs, revealed that the characteristic peaks of the drugs were predominantly replaced with the characteristic peaks of PLGA after encapsulation. In normal lung fibroblast, we also demonstrated the cytotoxicity of FTY720-NOB-PLGA NPs is less than free drug at a higher dose combination (Supplementary Figure 2). In summary, our findings suggest that FTY720-NOB-PLGA NPs served as effective drug carriers, successfully encapsulating both FTY720 and NOB. This dual encapsulation was further validated through encapsulation efficiency assay data (Supplementary Table 2 and Table 3). Notably, the drug release data (Figure 3B,C) revealed that both drugs can be continuously released for up to 5 days, underscoring the stable and slow-release functionality of FTY720-NOB-PLGA NPs. Our *in vitro* study demonstrated that FTY720-NOB pure drug and FTY720-NOB-PLGA NPs groups provided no significant difference in IL-6 inhibition at 24 and 72 h. It is possible that this is due to the fact that both the pure drug group and the NPs group were dissolved in Dimethyl sulfoxide (DMSO) for the cellular experiments, so the effects of both groups would be similar. This result also suggests that FTY720 and NOB are still effective after nanoencapsulation, and the synergistic impact of the dual drug was more potent in suppressing cytokine release than using a single drug alone (Figure 4B).

It is a matter of concern whether administering a non-nanoparticle formulation of the two drugs via inhalation would yield comparable efficacy upon repeated administration. To compare this to the benefits observed with nanoencapsulation, we conducted tests involving the administration of the free drugs in animal models. Since FTY720 contains hydrophilic and hydrophobic groups and NOB is lipophilic with poor solubility in water, DMSO was used as a cosolvent in an animal model. This free-drug regimen led to acute distress; two mice subjected to it died within a day, unlike their counterparts receiving the nanoencapsulated drug, who survived throughout the study. Histological analysis of the affected mice revealed suboptimal treatment efficacy (Supplementary Figure 7). The precise reasons behind the increased side effects of the free drug combination compared with the FTY720-NOB-PLGA NPs formulation remain unclear. Nonetheless, existing literature suggests that NPs-conjugated drugs show reduced toxicity compared to free drugs when delivered via inhalation.^33−35^ Subsequently, we examined lung tissue samples from mice treated with FTY720-NOB in its pure form and compared them with those treated with FTY720-NOB-PLGA NPs. Our analysis indicated that the anti-inflammatory effects were less pronounced in the pure drug group than that in the FTY720-NOB-PLGA NPs formulation. Pharmacokinetics of inhaled drugs in the lungs differ significantly from that of oral or intravenous administration. Lipophilic drugs are rapidly absorbed through passive cross-cellular diffusion into the epithelial cells upon dissolution. Alveolar absorption clearance is notably faster due to the extensive absorptive surface, thin epithelium, and pulmonary circulation supply. An ideal pulmonary drug delivery system should sustain drug release to account for delayed pulmonary clearance mechanisms and ensure effective lung deposition, thereby maintaining prolonged therapeutic drug concentrations. Therefore, researching these factors is crucial for improving drug bioavailability in pulmonary drug delivery systems.^36^ Moreover, we performed *in vivo* fluorescent PLGA-Cy5 NPs distribution and cellular uptake of fluorescent PLGA-Cy5 NPs to confirm that the nanoparticles reach the alveoli (Supplementary Figure 5A). This figure presents the distribution of fluorescent PLGA-Cy5 in the alveoli postinhalation *in vivo*. Additionally, delivery to the alveoli of the nanoparticles was observed under high magnification through confocal microscopy, as shown in Supplementary Figure 5B (white arrow). Further quantification was performed by comparing the red fluorescent light reaching the alveoli with the red fluorescent light initially delivered according to our previously published method,^37^ suggesting in a PLGA nanoparticle delivery rate to the alveoli of approximately 70%. Based on the accumulated inhaled aerosol (AIA), we could calculate that the concentration of the drug in the encapsulated form that reaches the epithelial cells of the alveoli dose is approximately FTY720 6.17 × 10^–5^ mg and NOB 3.185x 10^–4^ mg (shown in Supplementary Table 5).

Binding of LPS to the TLR4 receptor triggers the upregulation of proinflammatory mediators. When LPS stimulated Raw264.7 cells, they released cytokines. Our *in vitro* study demonstrated that FTY720-NOB-PLGA NPs exhibited a stronger inhibitory effect than the pure drug in reducing the expression of IL-6 and ROS induced by LPS. The synergistic impact of the dual drug was more potent in suppressing cytokine release than using a single drug alone (Figure 4). NF-κB expression in the LPS-induced group was confirmed through IHC staining of the lung tissue. After the NF-κB dimer degraded, it translocated to the nucleus, leading to the stimulation of IL-6 and TNF-α production. This, in turn, reactivated IκB kinase, which phosphorylated IκB, thus perpetuating the inflammatory response. Within the first 24 h of FTY720-NOB-PLGA NPs treatment, NF-κB expression significantly reduced. However, after 48 h of treatment, the translocation of the NF-κB dimer to the nucleus was unchanged, and the cytokine assay indicated that the inflammatory response was not alleviated (Figure 6). Therefore, triple doses of FTY720-NOB-PLGA NPs were necessary to effectively alleviate lung injury. It is important to note that FTY720-NOB-PLGA NPs inhibited LPS-induced p38 phosphorylation but did not affect JNK (Supplementary Figure 8), unlike another study showing that bacterial infection-induced lung injury activates NF-κB expression via the upstream pathways of p38 and JNK.^38^ In Supplementary Figure 6, we further demonstrated cellular uptake of FTY720-NOB-PLGA(Cy5) in distinct immune cell populations. And the inhalation of LPS was found to induce the influx of neutrophils and macrophages, a response that was effectively suppressed following inhalation of FTY720-NOB-PLGA NPs (Figure 8and Figure 9). Neutrophils are the initial responders to chemokines at sites of inflammation, including in acute lung injury, where they traverse the endothelial and epithelial barriers into the alveoli, releasing ROS and neutrophil extracellular traps that disrupt the endothelial–epithelial barrier. Studies have shown that macrophage depletion in response to Gram-negative bacterial endotoxin reduces NF-κB activation, cytokine production, and neutrophil influx.^39^ Macrophages play a crucial role in the defense of foreign particles and pathogens. During acute lung injury, macrophages release various pro-inflammatory cytokines, including IL-1β, IL-6, and TNF-α. Since nanoparticles can be easily phagocytosed by macrophages, they effectively modulate macrophage-mediated inflammatory responses.^40^ Research has highlighted the potential of nanomedicine in targeted macrophage therapy for ALI, particularly through regulating macrophage polarization.^20^ Our study elucidated the potential mechanism by which FTY720-NOB-PLGA NPs attenuate ALI, specifically by suppressing macrophage recruitment and reducing IL-6 and ROS secretion, thereby decreasing neutrophil accumulation.

The daily administration of inhaled FTY720-NOB-PLGA NPs demonstrates remarkable promise, with histological staining and the evaluation of NF-κB expression indicating no significant deviations from normal control levels. Unlike previous studies using tracheal installation for drug administration, our unique inhalation system for acute lung injury is equipped with an inhalation tower, enabling precise control over pressure, temperature, and humidity within the inhalation chamber while simultaneously monitoring changes in respiratory parameters during LPS exposure and inhalation treatment. Finally, our findings confirm that FTY720-NOB-PLGA NPs do not induce organ dysfunction, particularly in the liver and kidneys.

Our study has some limitations. First, we did not compare the anti-inflammatory effects of FTY720-PLGA NPs, NOB-PLGA NPs, and FTY720-NOB-PLGA NPs against those of FTY720-PLGA NPs, making it unclear whether the effects arise from FTY720, NOB, or their combination. FTY720-PLGA NPs demonstrated anti-SARS-CoV-2 activity at noncytotoxic concentrations, showing nearly 70 times greater efficacy in inhibiting viral infection compared to the free drug^41^ but its *in vivo* toxicity has not been assessed. Compared to FTY720-NOB-PLGA NPs, which reduce acute lung injury by limiting macrophage recruitment and lowering IL-6 and ROS without affecting T cells, FTY720 nanoparticles have demonstrated promise in treating Parkinson’s disease and enhancing postorgan transplantation survival through different immunomodulatory mechanisms. In a Parkinson’s model, FTY720-NPs injections reduced MPO and NOS activity, downregulated IL-6 and TNFα, and shifted microglia from M1 to M2 phenotype. They also limited effector T cell infiltration and stabilized regulatory T cells via PP2A/EZH2/FOXP3 signaling pathway, highlighting their neuroprotective effects.^42^ In a mouse heart transplantation model, lymph node-targeting FTY720@TNP injections effectively suppress macrophages activity, boost Tregs levels, reduce effector T cells in lymph nodes, and minimize systemic immune responses, significantly prolonging survival without the side effects commonly associated with traditional immunosuppressants.^43^ Our attempts to encapsulate FTY720-PLGA NPs were unsuccessful due to low encapsulation efficiency, possibly caused by methodological differences. Nonetheless, we confirmed the safety and efficacy of FTY720-NOB-PLGA NPs in reducing inflammation when inhaled.

On the other hand, the current study did not evaluate whether FTY720 administered via injection or orally for ALI produces effects similar to those of our nanoparticle formulations. Previous research examined the concentration-dependent effects of FTY720 on endothelial barrier function *in vitro* and its impact on lung injury caused by mechanical ventilation (MV) and hyperoxia in mice. FTY720 administration by injection showed protective effects on human umbilical vein endothelial cell (HUVEC) barriers at concentrations up to 1 μM, while concentrations of 100 μM resulted in irreversible barrier breakdown and increased apoptosis. Low doses of FTY720 (0.1 mg/kg) decreased lung permeability in ventilated mice, whereas a higher dose (2 mg/kg) increased pulmonary vascular permeability and endothelial apoptosis in these animals.^44^ In a bleomycin-induced lung injury model treated with FTY720 via intraperitoneal injection, prolonged exposure was found to markedly exacerbate vascular leakage. Long-term exposure may lead to functional antagonism of the sphingosine-1-phosphate receptor 1 (S1P1), impairing its ability to maintain barrier integrity and promoting fibrosis, ultimately increasing mortality.^45^ Recent studies have elucidated the mechanisms involved; intraperitoneal injection of free FTY720 significantly improved lipopolysaccharide-induced acute lung injury at low doses (0.1, 0.2, and 0.5 mg/kg) by reducing proinflammatory cytokines (TNF-α, IL-6, and IL-1β), inhibiting NF-κB activation, and protecting the pulmonary capillary barrier. However, the inhibitory effect was diminished in higher dose groups (1 and 2 mg/kg).^11^ Since the treatment strategy for ALI should address key factors such as adequate dosage, sustained efficacy, and drug safety, it appears that free FTY720 administered via injection does not adequately meet these criteria. Similar to findings from other studies that highlight the advantages of encapsulated FTY720,^46−48^ the nanoparticle drug delivery system in the current study demonstrated prolonged protective effects (Figure 7) with enhanced safety, without inducing organ dysfunction or respiratory distress (Figure 10), thus offering promising therapeutic potential for the treatment of ALI.

## 4 Conclusions

Our study demonstrates the potential of inhaled FTY720-NOB-PLGA NPs as a promising treatment for ALI. These nanoparticles can be effectively delivered to the lungs and reach the lower airways. They exhibit a synergistic effect in reducing lung injury, suppressing inflammatory mediators, and inhibiting key components of the ALI pathway and do so without adverse effects on lung function or biochemical indicators. Our noninvasive inhalation approach offers a safe and efficient means of treating ALI, and these aerosolized NPs hold great promise for the future of ALI treatment.

## 5 Methods and Experiments

### 5.1 Chemicals and Reagents

FTY720 was purchased from TargetMol Chemicals Inc. (USA). NOB was provided by Prof. Min-Hsiung Pan from the Institute of Food Science and Technology, National Taiwan University. PLGA was obtained from Evonik Industries AG (Germany). Cellu-SepT4 dialysis bag was purchased from Membrane Filtration Products, Inc. (USA). IMR-90 and Raw264.7 cell lines were purchased from the Bioresource Collection and Research Center (Taiwan). LPS from the *Escherichia coli* serotype (O55:B5) was purchased from Sigma-Aldrich (USA). Cell counting kit-8 (CCK-8 kit) was purchased from Dojindo Molecular Technologies, Inc. (Japan). ROS-GloTM assay was purchased from Promega (USA). Mouse interleukin 6 ELISA kit was purchased from Elabscience (USA). TNF-α mouse Instant ELISA kit was purchased from Thermo Fisher Scientific Inc. (USA). Nuclear factor κB p65 antibody (NF-κB antibody) was purchased from GeneTex (USA). DAB substrate kit was purchased from Vector Laboratories Inc. (USA). Peroxidase-conjugated affiniPure goat anti-rabbit IgG (H+L) was purchased from Jackson ImmunoResearch Laboratories Inc. (USA). Anti-p-p38 antibody, anti-p-JNK antibody, anti-p38 antibody, and anti-JNK antibody were purchased from Cell Signaling Technology Inc. (USA).

### 5.2 Methods

#### 5.2.1 Synthesis of Inhalational FTY720-NOB-PLGA NPs

Inhaled FTY720-NOB-PLGA NPs were prepared by using the emulsion solvent evaporation technique. Initially, 100 mg of PLGA (50:50 copolymer composition; MW 64 kDa) was dissolved in 2.5 mL of dichloromethane (DCM) within an ice bath. Subsequently, 5 mg of FTY720 and 20 mg of NOB were dissolved in 1 mL of DCM and added to the PLGA solution. The resulting mixture was sonicated at 60% amplitude for 2 min using a probe sonicator. A 1% w/v polyvinyl alcohol (PVA) aqueous solution (MW 30–70 kDa) was introduced to the oil phase, and the emulsion was sonicated again at 60% amplitude for 2 min. The final emulsified solution was transferred to a double-ended round-bottomed flask and placed in a 75 °C water bath with constant stirring at 400 rpm under a vacuum to facilitate complete evaporation of the organic solvent. The synthesized emulsion was then centrifuged at 10 000 rpm for 10 min using a high-speed microrefrigerated centrifuge. The supernatant was collected, and the FTY720-NOB-PLGA NPs in the centrifuge tube could be washed with deionized water or PBS. A schematic diagram of the synthesis process was provided in Supplementary Figure 9.

#### 5.2.2 Characterization of Inhalational FTY720-NOB-PLGA NPs

The particle sizes and ζ potentials of the FTY720-NOB-PLGA NPs were determined by using a DelsaNano instrument (Beckman Coulter Inc., USA). All samples were diluted 100 times with deionized water, and measurements were conducted in triplicate at 25 °C. Additionally, the prepared samples were examined with a JEM-1400 transmission electron microscope (TEM) (JEOL, USA). Chemical analysis of functional groups was performed using an FT/IR-4600 Fourier transform infrared spectrometer (FTIR) (Jasco, Japan). The material was freeze-dried into a powder and placed on a disc stage for measurement within the range of 500–4000 cm^–1^ at a resolution of 0.7 cm^–1^.

#### 5.2.3 Encapsulation Efficiency of Inhalational FTY720-NOB-PLGA NPs

FTY720 and NOB concentrations within PLGA were determined via UV spectroscopy. The supernatant, after dilution with deionized water, was assessed for FTY720 absorbance at 200 nm using a NanoDrop Onec. For FTY720-NOB-PLGA NPs, 100 μL of the centrifuged solution was treated with 1 mL of DMSO for uniform drug dispersion via an ultrasonic vibration. The absorption value of the NOB was quantified at a wavelength of 330 nm. The UV absorbance data were employed to calculate the concentrations of FTY720 and NOB using standard curves. Equations 1 and 2 were employed to determine drug encapsulation efficiency (EE) %, representing the drug content as a percentage of the initial drug amount, and drug loading efficiency (DL) %, indicating the drug content as a percentage of the initial polymer amount.

Equation 1 shows the formula for drug encapsulation efficiency (EE), %:
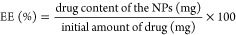
1Equation 2 shows the formula for drug loading efficacy (DL), %:
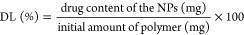
2

#### 5.2.4 *In Vitro* Accumulated Drug Release

Two-milliliter volumes of the aqueous solution containing FTY720-NOB-PLGA NPs were packed into CelluSepT4 dialysis membranes (MW: 12000–14000) and immersed in 18 mL of PBST at pH 7.4 to investigate the sustained release dynamics of FTY720-NOB-PLGA NPs. These samples underwent gentle agitation at 37 °C. At specified time intervals, the release solution samples were withdrawn, and 18 mL of fresh PBST was introduced. The absorption values of FTY720 and NOB were determined using NanoDrop Onec at their respective drug-specific λ_max_. All experiments were conducted in triplicate, and the results were averaged. The drug release efficiency (RE), %, was calculated as the percentage of the released drug relative to the drug content of FTY720-NOB-PLGA NPs, as defined in eq 3. Equation 3 shows the formula for drug releasing efficiency (R.E.) %
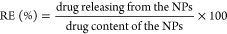
3

#### 5.2.5 Cell Viability Assays

Human lung fibroblast cells (IMR-90) were cultured at 37 °C in an environment with 90% relative humidity and 5% CO_2_ in minimum essential medium (MEM) containing 0.1 mM nonessential amino acids (NEAA), 1.0 mM sodium pyruvate, 10% (v/v) fetal bovine serum (FBS), and antibiotics. Cytotoxicity was assessed using a cell counting kit-8 (CCK-8). IMR-90 cells (5 × 10^3^ cells per well in 96-well plates) were exposed to varying drug concentrations for 24 and 48 h. Subsequently, 10 μL of a CCK-8 solution was added to the medium. After a 2 h incubation, cell viability was determined by measuring the absorbance at 450 nm with a multiplate reader (DeTie, China).

#### 5.2.6 Interleukin-6 Assay

Raw264 cells were cultured at 5 × 10^3^ cells per well for a 96-well plate. In each well, 10 μg/mL of LPS was added to induce inflammation in cells for 24 h, and different concentrations of drugs were added and incubated for 24, 48, and 72 h. To use the mouse IL-6 ELISA kit purchased by Elabscience Biotechnology Inc., add 100 μL of testing cell suspension to each well before incubating for 90 min at 37 °C. Then, we decanted the liquid from each well and added 100 μL of biotinylated detection Ab working solution to each well to incubate for 1 h. After 350 μL of wash buffer was added for washing, it was repeated three times. We added 100 μL of HRP conjugate working solution to wait 30 min and 90 μL of substrate reagent for 15 min. Finally, we added 50 μL of stop solution and determined the OD value of each well at once with a microplate reader set to 450 nm.

#### 5.2.7 Measurement of Total ROS

Raw 264.7 cells were cultured in Dulbecco’s modified Eagle’s medium supplemented with 10% (v/v) FBS at 37 °C under 90% relative humidity and 5% CO_2_. Total ROS were measured by adding a detection reagent that contained Ultra-Glo recombinant luciferase and d-cysteine. The cells were cultured at a density of 1 × 10^4^ cells per well in a 96-well plate. In each well, 10 μg/mL of LPS was introduced to initiate cell inflammation for 24 h. Different concentrations of the drug were added and allowed to stand for 24 h. Six hours before the end of the experiment, we added 20 μL of H_2_O_2_ substrate reagent followed by 100 μL of ROS-Glo detection reagent containing Ultra-Glo recombinant luciferase and d-cysteine to a 96-well plate 20 min before measurement. The signal was detected by the Glomax 20/20 luminometer (Promega, USA).

#### 5.2.8 Animals’ Studies

All laboratory animal projects conducted at National Cheng Kung University received approval from the Institutional Animal Care and Use Committee (IACUC No. 112057). The male BALB/c mice used in this study were between 10 to 12 weeks old and weighing 20–25 g. BioLASCO Taiwan Co., Ltd. provided the mice, which were housed under standard conditions on a 12 h light/12 h dark cycle in sterile cages. Food and water were freely available. After a week of acclimation to their adapted environment, the experiments began.

#### 5.2.9 Animal Models for the Introduction of ALI

Male BALB/c mice were randomly assigned to one of four groups, with three mice in each group: (i) control group (PBS), (ii) LPS/PBS group, (iii) LPS/NOB-PLGA group, and (iv) LPS/FTY720-NOB-PLGA group. To induce ALI, mice were anesthetized and then given 30 mg/kg of LPS through intratracheal instillation. The control group received sterile PBS instead.

#### 5.2.10 Nose-Only Inhalation Tower System

The inhalation tower features seven open ports. Following a 1 h LPS induction, mice were placed within a nose-only Allay restrainer in the inhalation chamber, ensuring proper alignment to avoid thoracic compression and maintain normal breathing. Inflows and pressures were set at 0.5 L/min per port and −0.5 cmH_2_O, respectively. A approximate 4 μm particle-size drug solution (comprising PBS, NOB-PLGA NPs, and FTY720-NOB-PLGA NPs) was uniformly nebulized across all tower ports. Reference dosage information for FTY720 and NOB is available in Supplementary Table 5. Continuous monitoring of the inhalation tower’s environmental conditions was enabled through a temperature and humidity probe inserted into one of its ports, controlled by specialized software that defined temperature, humidity, O_2_/CO_2_ ratios, flow rates, and pressure. Respiratory parameters of the mice during drug administration were quantified via plethysmography. Additionally, daily assessments included monitoring the body weight, physiological status, and overall vitality.

#### 5.2.11 Confocal Microscope Image of Lung Deposition and Cellular Uptake of PLGA-Cy5, IHC Staining for NF-κB, Immune Cell and Multiplexed mIF Assay

To synthesize fluorescent PLGA-Cy5 nanoparticles,^49^ PLGA-Cy5 (50 mg) was dissolved in 2.5 mL dichloromethane and then in 1 mL ethanol, followed by sonication at 130 W for 2 min to achieve a W1/O emulsion. Addition of 10 mL of 1% PVA and a further 2 min of sonication formed a W1/O/W2 emulsion, all conducted at 4 °C. The resulting mixture was centrifuged at 10 000 rpm for 15 min at 4 °C to yield pellets, which were then resuspended in 3 mL of PBS. For *in vivo* lung distribution and cellular uptake assessment, fluorescent PLGA-Cy5 at a concentration of 20 mg/mL, diluted in saline to a total volume of 2.5 mL, was administered via an inhalation tower system. Mouse lung tissues were sectioned into 14 μm slices, stained with Cell Mask Green for the membrane and DAPI for nuclei. Cy5-PLGA was sourced from CD-Bioparticles, while Cell Mask Green and DAPI were obtained from Thermo Fisher and Life Technologies, respectively. Imaging was performed using an Olympus FV-3000 confocal microscope.

For IHC staining, the lung tissue underwent fixation in 4% paraformaldehyde (in PBS) overnight, followed by embedding in paraffin wax. Subsequently, sections were stained with H&E and examined using an Olympus BX51 optical microscope (Olympus, Japan). IHC staining for NF-κB (GeneTex, 102090) was performed on the lung slices, and quantification was carried out using TissueQuest software. This analysis aimed to confirm the anti-inflammatory and ALI-reversal effects.

To evaluate immune cell recruitment after LPS stimulation with or without NPs inhalation treatment, neutrophils, macrophages, and T cells were quantified by manually counting cells positively labeled with specific antibodies across 10 high-power fields (HPF) per slide. IHC staining was performed using the following primary antibodies: rabbit antimyeloperoxidase (Abcam, ab9535) for neutrophils, rabbit anti-CD68 (Abcam, ab303565) for macrophages, and rabbit anti-CD3 (GeneTex, GTX16669) for T cells. All immunostaining analyses were conducted by a pathologist (Chung-Ta Lee) who was unaware of the inhalation treatments administered to the mice.

To simultaneously detect multiple immune cell markers within the same tissue section, a multiplexed mIF assay was conducted using the Opal 6-Plex detection kit (AKOYA #811001, USA).^22^ Primary antibodies for myeloperoxidase (Abcam, ab9535, 1:50), CD68 (Abcam, ab303565, 1:4000), and CD3 (GeneTex, GTX16669, 1:25) were incubated at room temperature for 2 h. Subsequently, samples were incubated at 37 °C for 10 min with a secondary antibody using the Opal polymer anti-rabbit horseradish peroxidase (HRP). The Opal 6-Plex detection kit was employed to visualize the tyramide signal amplification (TSA) using Opal dyes 520, 570, 620, and 690. Finally, nuclei were counterstained with DAPI (1:100) for 10 min. Image acquisition was performed by using TissueFAXS (TissueGnostics).

#### 5.2.12 Assay of Inflammatory Mediator Levels in Bronchoalveolar Lavage Fluid

BALF was collected by performing a tracheostomy, followed by insertion of a needle into the trachea. After the mice were sacrificed, the lungs underwent three lavages with 1 mL of sterile PBS. The resulting BALF was collected into tubes. Subsequently, BALF samples were centrifuged at 1000*g* for 20 min at 4 °C, and the supernatant was obtained for the measurement of IL-6 and TNF-α concentrations using an ELISA kit.

#### 5.2.13 Western Blot

Lung tissue proteins were extracted using lysis buffer (RIPA with protease and phosphatase inhibitor) on ice for 30 min. Protein concentrations were assessed with a BCA protein assay kit (BIO-RAD). Subsequently, samples were separated by 10% SDS–PAGE and transferred to polyvinylidene difluoride membranes. Following blocking with 5% nonfat milk, the membranes underwent incubation with primary antibodies, including Anti-p-p38 (Cell Signaling, 4511), Anti-p-JNK (Cell Signaling, 9255), Anti-p38 (Cell Signaling, 8690), and Anti-JNK (Cell Signaling, 9252). This was followed by incubation with the conjugated secondary antibodies. Immunodetection was carried out using an enhanced chemiluminescence detection kit. Actin (Millipore, MAB1501) served as an internal control for protein loading.

#### 5.2.14 Statistical Analysis

Data are expressed as mean ± SD (standard deviation), with representative outcomes from independent experiments, each performed in triplicate. Statistical differences between groups were assessed using analysis of variance (ANOVA) and the *t* test. A *p*-value less than 0.05 was considered indicative of a significant difference, while a *p*-value less than 0.01 was deemed highly significant. Furthermore, a *p*-value less than 0.001 was considered as indicative of a very high level of significance.

## Supporting Information Available

Referenced data . The Supporting Information is available free of charge at https://pubs.acs.org/doi/10.1021/acsnano.3c12532.

Tables S1–S5 and Figures S1–S9 providing comprehensive experimental information; emulsion solvent evaporation method for synthesizing inhaled FTY720-NOB-PLGA NPs; reference dosage information for FTY720 and NOB; particle number distribution, polydispersity index, and ζ potential of FTY720-NOB-PLGA NPs; drug concentrations of FTY720 and NOB after encapsulation in PLGA; concentrations of NOB postrelease from NOB-PLGA NPs; viability of normal lung fibroblasts treated with free drug or PLGA-encapsulated nanoparticles; LPS-induced IL-6 secretion in normal lung fibroblast cells; inhalation tower setup and pulmonary function parameters under various treatments; distribution of nanoparticles in alveolar regions and their uptake by distinct immune cell populations observed via confocal microscopy (PDF)
