# Supplementary material for: Enhancing Lung Recovery: Inhaled Poly(lactic-co-glycolic) Acid Encapsulating FTY720 and Nobiletin for Lipopolysaccharide-Induced Lung Injury, with Advanced Inhalation Tower Technology
Source: ACS Nano. 2025 Feb 18;19(8):7634–49. doi: 10.1021/acsnano.3c12532 (PMC11887484; doi:10.1021/acsnano.3c12532)
Supplement: Supplementary file 1 — nn3c12532_si_001.pdf [file nn3c12532_si_001.pdf]

Supporting Information

Enhancing Lung Recovery: Inhaled poly(lactic-co-glycolic) acid Encapsulating FTY720 and Nobiletin for Lipopolysaccharide-Induced Lung Injury, with Advanced Inhalation Tower Technology

Huei-Han Zhang [a,1], Wen-Shuo Kuo [b,1], Pei-Yu Tu [a], Chung-Ta Lee [c], Hao-Chen Wang [d], Yu-Ting Huang [e], Mei-Chun Shen [e], Tsai-Shiuan Lin [e], Po-Lan Su [e], Jeng-Shiuan Tsai [e,f], Min-Hsiung Pan [g,h], Chien-Chung Lin [e,f,i,j], Ping-Ching Wu [a,k,,l,m*].*

[a] Department of Biomedical Engineering, National Cheng Kung University, Tainan 70101, Taiwan

[b] Center for Allergy Immunology and Microbiome (AIM), China Medical University Children's Hospital/China Medical University Hospital, China Medical University, Taichung 404327, Taiwan

[c] Department of Pathology, National Cheng Kung University Hospital, College of Medicine, National Cheng Kung University, Tainan 701401, Taiwan

[d] Center of Comparative Medicine and Research, Innovation Headquarters, National Cheng Kung University, Tainan 70101, Taiwan

[e] Department of Internal Medicine, National Cheng Kung University Hospital, College of Medicine, National Cheng Kung University, Tainan 70403, Taiwan

[f] Graduate Institute of Clinical Medicine, College of Medicine, National Cheng Kung University, Tainan 701401, Taiwan

[g] Institute of Food Science and Technology, National Taiwan University, Taipei 10617, Taiwan

[h] Department of Medical Research, China Medical University Hospital, China Medical University, Taichung 404327, Taiwan

[i] Tainan Hospital, Ministry of Health & Welfare, Tainan 700, Taiwan.

[j] Institute of Molecular Medicine, College of Medicine, National Cheng Kung University, Tainan 70101, Taiwan

[k] Center of Applied Nanomedicine, National Cheng Kung University, Tainan 70101, Taiwan

[l] Medical Device Innovation Center, Taiwan Innovation Center of Medical Devices and Technology, National Cheng Kung University Hospital, National Cheng Kung University, Tainan 70403, Taiwan.

[m] University Center for Bioscience and Biotechnology, National Cheng Kung University, Tainan 70101, Taiwan.

[1] These authors contributed this work equally

*Correspondence to:

Ping-Ching Wu − Department of Biomedical Engineering, National Cheng Kung University, Tainan 70101, Taiwan; Center of Applied Nanomedicine, National Cheng Kung University, Tainan 70101, Taiwan; Medical Device Innovation Center, Taiwan; Innovation Center of Medical Devices and Technology, National Cheng Kung University Hospital, National Cheng Kung University, Tainan 70403, Taiwan; University Center for Bioscience and Biotechnology, National Cheng Kung University, Tainan 70101, Taiwan 0000-0001-6667-3492; Email: wbcxyz@bme.ncku.edu.tw

Chien-Chung Lin − Department of Internal Medicine, National Cheng Kung University Hospital, College of Medicine, National Cheng Kung University, Tainan 70403, Taiwan; Graduate Institute of Clinical Medicine, College of Medicine, National Cheng Kung University, Tainan 70101, Taiwan; Tainan Hospital, Ministry of Health & Welfare, Tainan 70101, Taiwan; Institute of Molecular Medicine, College of Medicine, National Cheng Kung University, Tainan 700, Taiwan; 0000-0002-4739-5631; Email: joshcclin@gmail.com

Supplementary Table 1. The number distribution, polydispersity index (P.I.), and zeta potential of co-encapsulated fingolimod (FTY720) and nobiletin (NOB) inhaled nanoparticles were prepared by the emulsification method using poly(lactic-co-glycolic) acid (PLGA).

	Size (nm)	Polydispersity Index	Zeta (mV)
FTY720-NOB-PLGA nanoparticles (NPs)	128.2	0.302	-9.08
	120.9	0.251	-10.88
	122.9	0.2	-13.58
	131.8	0.193	-12.11
	121.7	0.148	-14.77
	128.1	0.205	-14.45
	132.2	0.188	-15
	121.3	0.229	-16.65
	131.2	0.198	-15.42
Mean	126.5	0.213	-13.55

Supplementary Table 2. Drug concentration of FTY720 after encapsulating in PLGA

	gram (mg)	Encapsulation efficiency (%)	Drug loading efficacy (%)
FTY720	2.91	58.14	2.91
	2.82	56.44	2.82
	2.95	59.02	2.95
Mean	2.89	57.87	2.89

Supplementary Table 3. Drug concentration of NOB after encapsulating in PLGA

	gram (mg)	Encapsulation efficiency (%)	Drug loading efficacy (%)
NOB	14.90	74.50	14.90
	15.06	75.30	15.06
	14.85	74.25	14.85
Mean	14.93	74.68	14.93

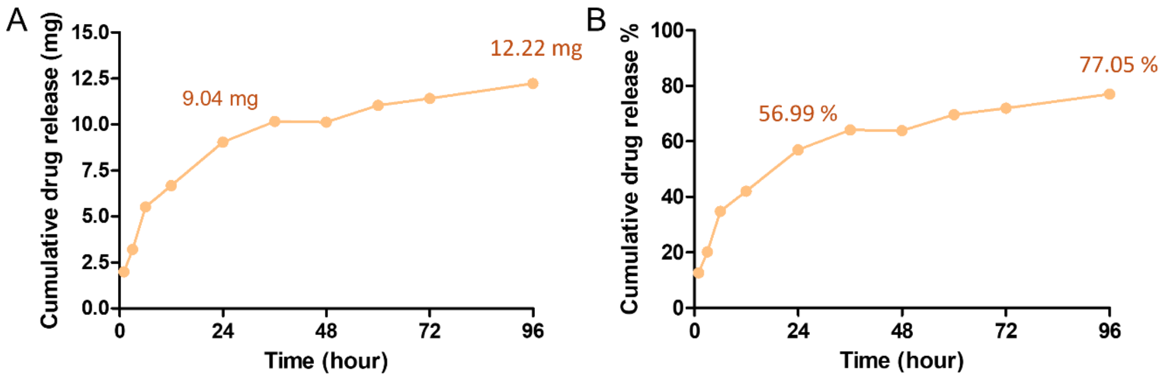

Supplementary Figure 1. The NOB-PLGA NPs underwent drug release at 1 x phosphate-buffered saline with Tween-20 (PBST) and 37°C. Concentrations of NOB were determined at wavelengths of 330 nm and expressed as (A) grams of cumulative drug and (B) percent of total cumulative drug.

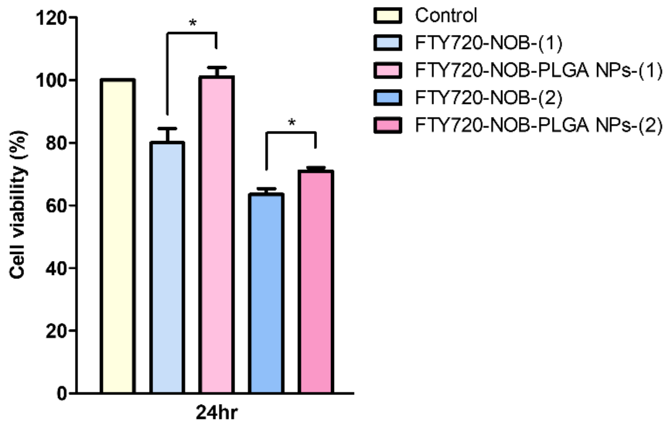

Supplementary Figure 2. Normal lung fibroblasts (IMR-90 cells) were treated with pure drug combinations (FTY20-NOB-(1) 8.125 x 10^{-4} mg/mL FTY20 + 5 x 10^{-3} mg/mL NOB and FTY20-NOB-(2) at 1.625 x 10^{-3} mg/mL FTY20 + 10^{-2} mg/mL NOB). Viability was assessed using the CCK-8 assay. Values are presented as means ± SD from three independent experiments. p-values were determined using the Student's t-test (*$p < 0.05$).

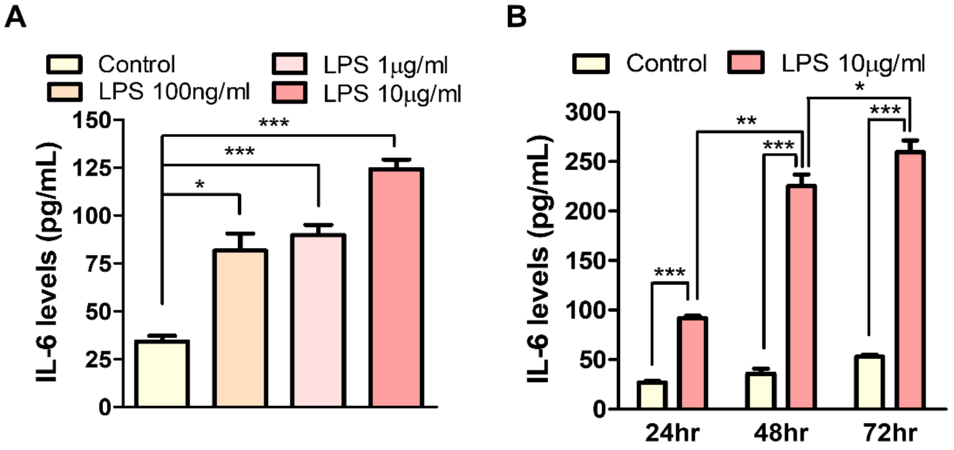

Supplementary Figure 3. Analysis of interleukin-6 (IL-6) secretion by Raw264.7 cells in response to (A) varying concentrations of lipopolysaccharide (LPS) and (B) stimulation with 10 μg/mL of LPS at different time points. Mean ± SD values are presented, and p-values were

determined using the Student's t-test. (***p < 0.001, **p < 0.01 and *p < 0.05).

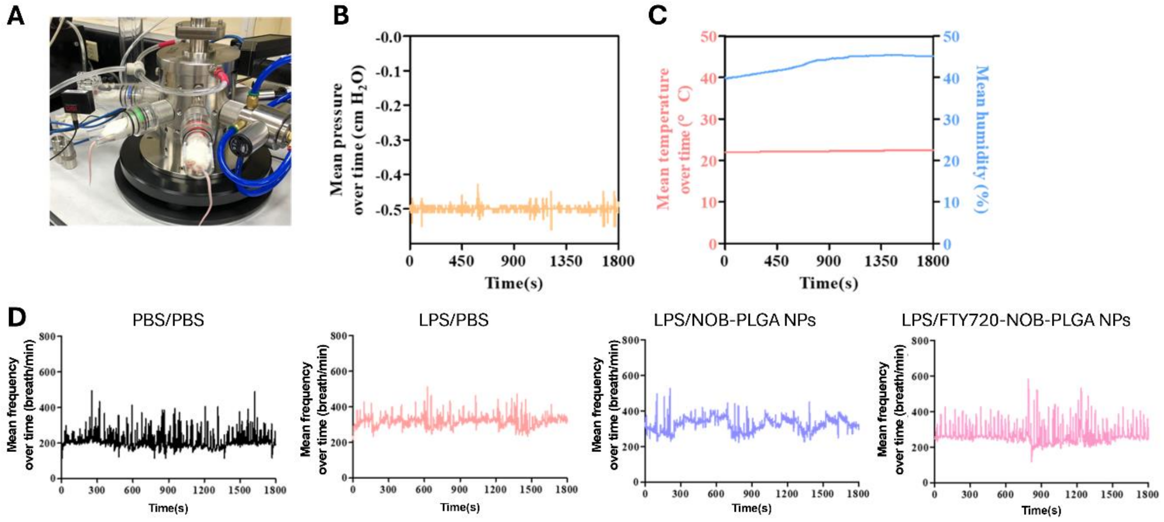

Supplementary Figure 4. (A)Photograph of the inhalation tower. (B) Mean of the pressure (cm H_2O) measured within the outer core of the nose-only inhalation tower during the exposure time. (C) Mean temperature (°C) and mean humidity over time at a chamber port during the exposure time. (D) Respiratory parameters such as mean breathing frequency were recorded during exposure through plethysmography during mouse exposure to PBS, LPS, NOB-PLGA NPs, and FTY720-NOB-PLGA NPs.

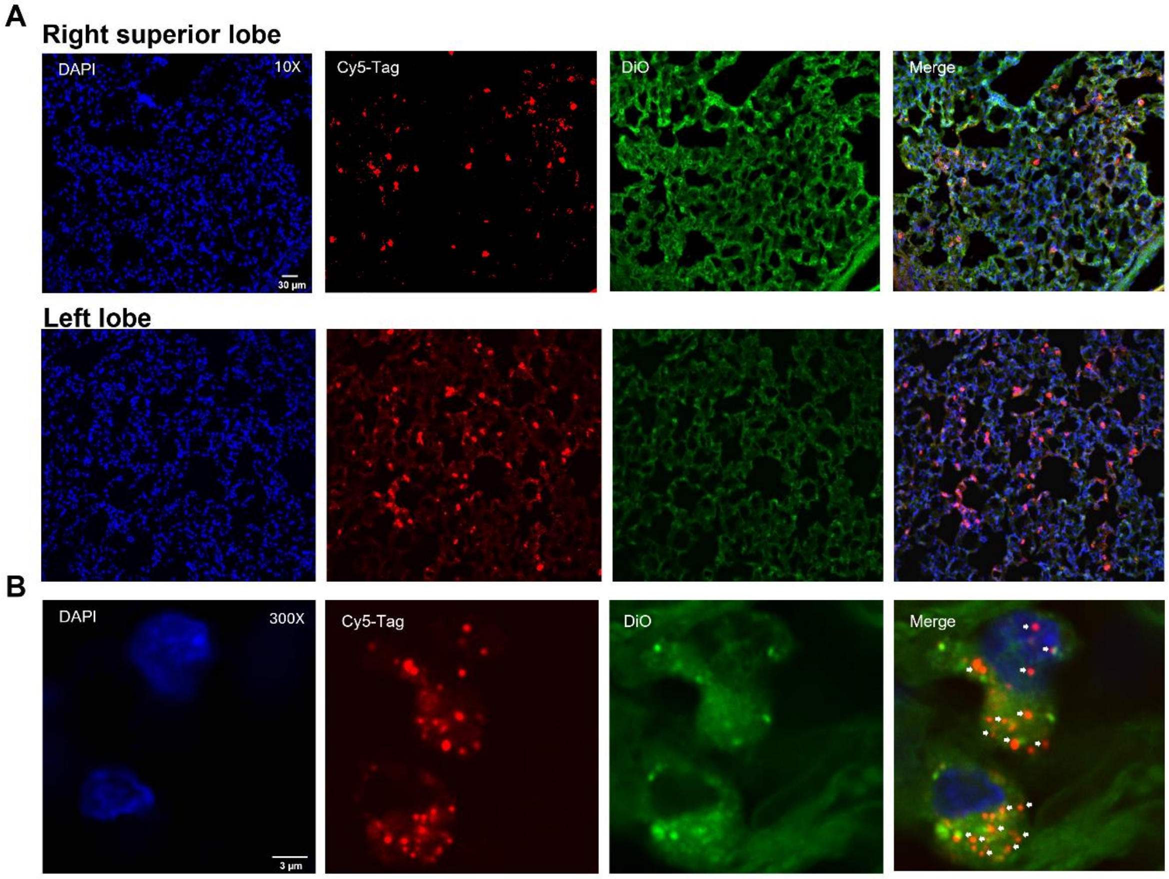

Supplementary Figure 5. (A) Confocal microscopy image displays the distribution of PLGA-Cy5 NPs (red) within the alveolar regions of the mouse lung, specifically the right superior and left lobes, 24 h following administration via an inhalation tower (magnification 20X). (B) A

detailed view of intracellular uptake of PLGA-Cy5 NPs (indicated by white arrows) within lung alveoli cells. Cell nuclei are counterstained with DAPI (blue), and the cell membrane is visualized using Cell Mask (green) (magnification 100X)

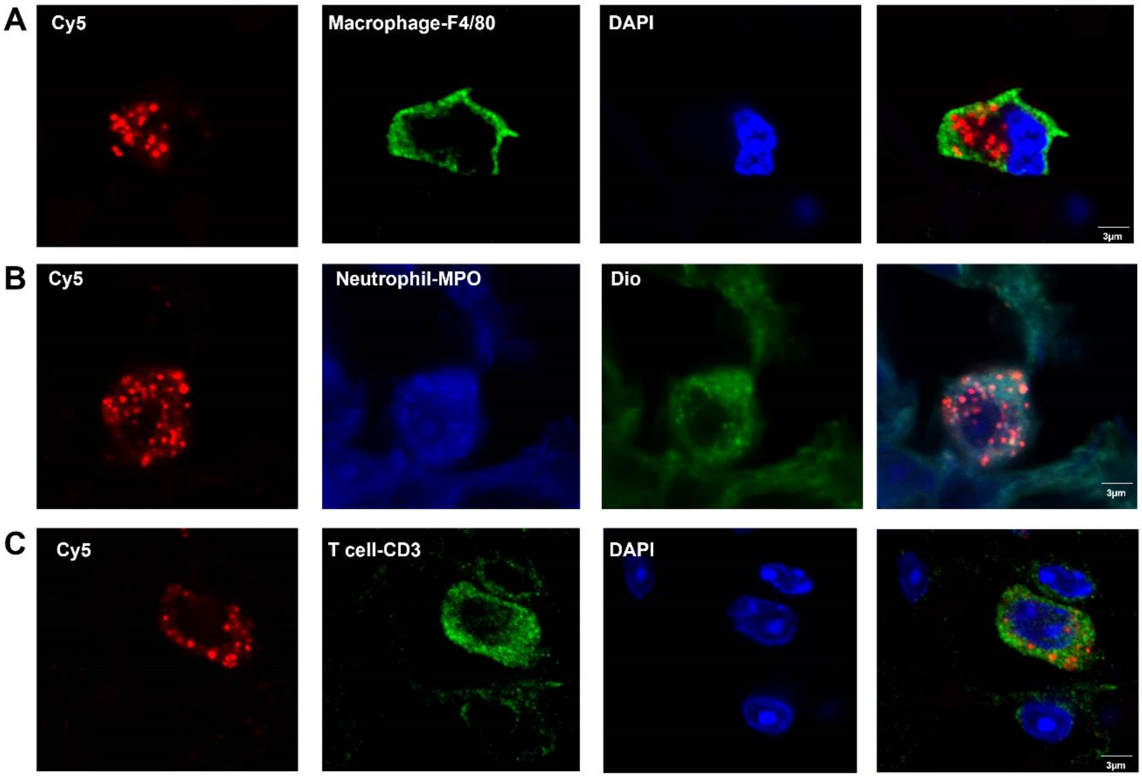

Supplementary Figure 6. Confocal microscopy image shows the distribution of FTY720-NOB-PLGA-Cy5 NPs (red) within different immune cells: (A) Intracellular uptake of FTY720-NOB-PLGA-Cy5 NPs in macrophages (F4/80, green) with nuclei stained with DAPI (blue). (B) Intracellular uptake of FTY720-NOB-PLGA-Cy5 NPs in neutrophils (myeloperoxidase - MPO, blue) showing cytoplasm colocalization with DIO (green). (C) Uptake of FTY720-NOB-PLGA-Cy5 NPs in T cells (CD3, green) with nuclei stained with DAPI (blue).

Supplementary Table 4. Investigating different formulations of FTY720-NOB-PLGA NPs

**FTY720 (mg)**	**NOB (mg)**	**PLGA (mg)**	**Size (nm)**	**Polydispersity Index**	**Zeta (mV)**	**Encapsulation efficiency of FTY720 (%)**	**Encapsulation efficiency of NOB (%)**
60	20	100	47.6	0.178	+31.37	4.6	68.1
20	20	100	111.9	0.195	+6.42	14.3	20 .0
5	20	100	126.5	0.213	-13.55	57.8	74.7

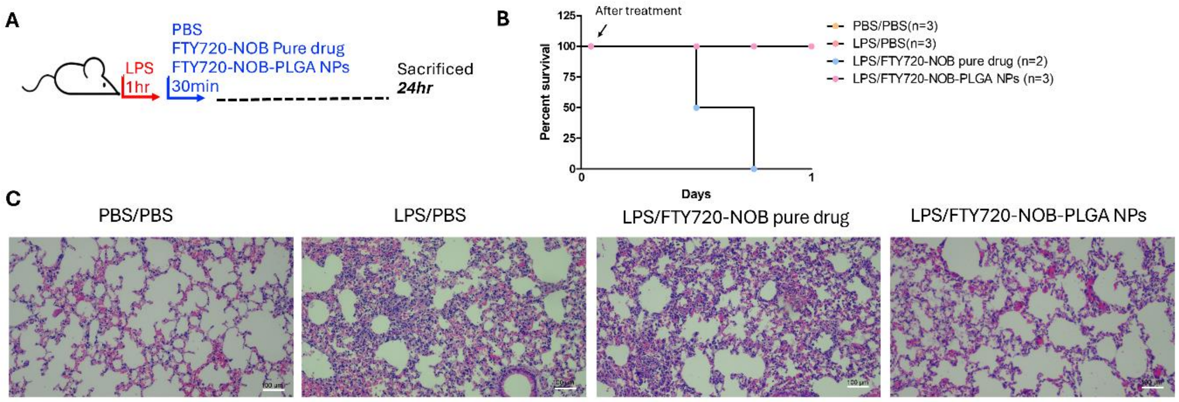

Supplementary Figure 7. (A) Schematic diagram of the animal model examining the endurance of FTY720-NOB pure drug and FTY720-NOB-PLGA NPs' efficacy over a 24 h period. (B) Survival rate of LPS-induced lung injury mice treat with PBS, FTY720-NOB free drug, and FTY720-NOB-PLGA NPs for 24 h. (C) Lung pathology evaluation in mice (n = 3 per group, except free drug) with LPS-induced lung injury treated with different formulation at 24 h, displayed at an original magnification of 100x.

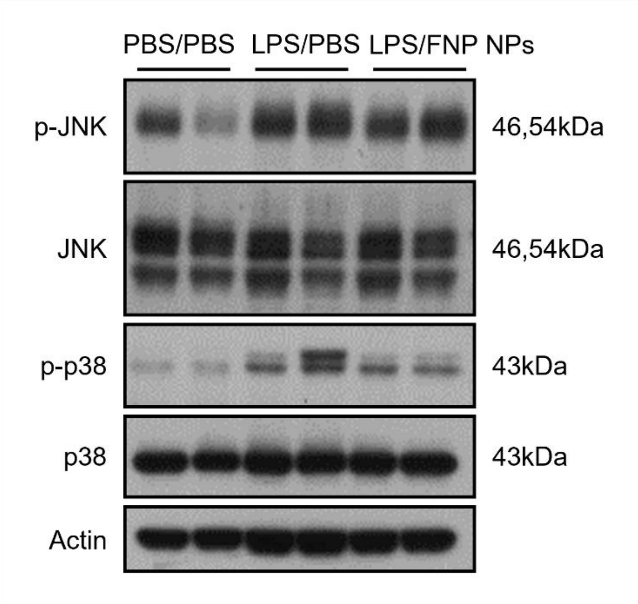

Supplementary Figure 8. To assess the capacity of FTY720-NOB-PLGA NPs to inhibit mitogen-activated protein kinases (MAPKs) in the lungs of mice with LPS-induced acute lung injury (ALI), Western blot analysis was performed for the phosphorylation of p38 and c-Jun N-terminal kinases (JNK).

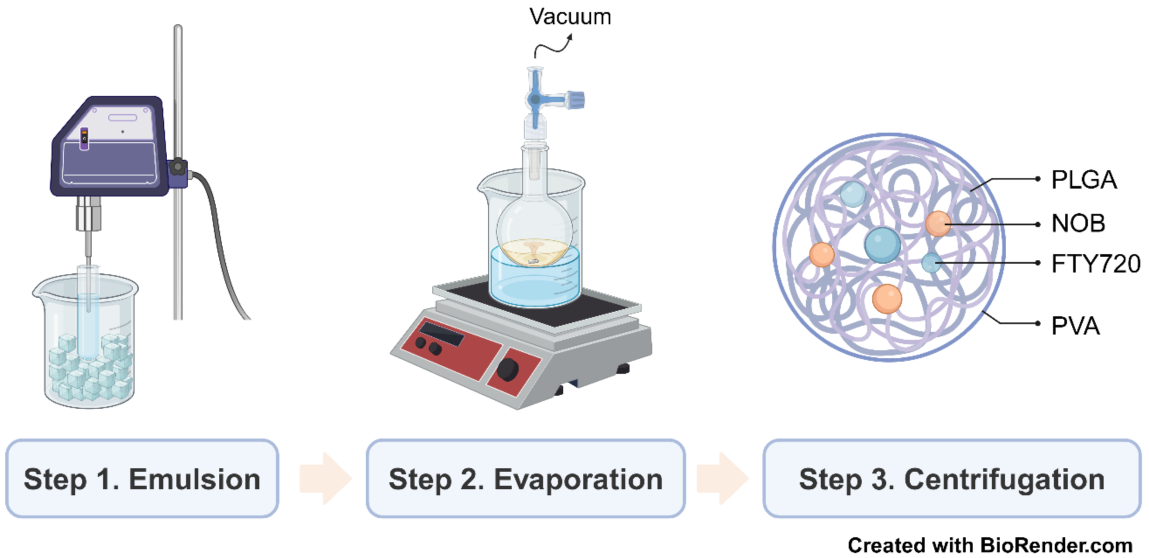

Supplementary Figure 9. Schematic diagram of the emulsion solvent evaporation method for the synthesis of inhaled FTY720- NOB- PLGA NPs.

Supplementary Table 5. The concentrations of drugs inhaled by mice through inhalation towers

	**AIA (mg)**	**Inhaled total dose in alveoli**
NOB-PLGA NPs	0.0292	3.14×10^{-4} mg (NOB)
FTY720-NOB-PLGA NPs	0.0299	6.17×10^{-5} mg (FTY720)<br>3.19×10^{-4} mg (NOB)
